# Comprehensive immune profiling reveals IFN-γ signaling in T cells mediates parasite phagocytosis in a rodent malaria model

**DOI:** 10.1128/mbio.03938-25

**Published:** 2026-03-11

**Authors:** Sha-sha Chen, Qingxin Yang, Yu Zhong, Defu Liu, Lifang Zhou, Han-cheng Wei, Chang-ling Li, Junwei Song, Yu-hang Wang, Xiao Hu, Lu Chen, Jing-wen Lin

**Affiliations:** 1 Center of Infectious Diseases and State Key Laboratory of Biotherapy, West China Hospital, Sichuan University 12530, Chengdu, Sichuan, China; 2 Biosafety laboratory of West China Hospital, West China Hospital, Sichuan University, Chengdu, Sichuan, China; 3 Key Laboratory of Birth Defects and Related Diseases of Women and Children of MOE, Department of Laboratory Medicine, West China Second University Hospital, Sichuan University 198150 , Chengdu, China; Universidad Complutense de Madrid, Madrid, Spain

**Keywords:** malaria, *Plasmodium*, lung pathology, CD8^+ ^T cell, T cell-monocyte interaction, CD8^+^Ly6C^+^ monocyte, phagocytosis

## Abstract

Pulmonary manifestation in malaria can be life-threatening. Using a rodent malaria model and comprehensive transcriptomics analyses to illustrate the immune landscape of malaria-associated lung pathology, we identified that IFN-γ signaling in T cells plays an important role in malaria-associated lung pathology. Disruption of IFN-γ signaling in T cells leads to reduced parasite load in the lungs and attenuated lung pathology by enhancing T cell-monocyte interaction. Surprisingly, the stronger interaction leads to an increase in a proinflammatory monocyte subset characterized by CD8 and Ly6C expression, which exhibits prominently elevated phagocytic capacity compared to the CD8^−^ counterpart. Our study illustrates the importance of IFN-γ signaling in T cell-monocyte interaction in malaria-associated lung pathology, highlighting the complex and intricate immune network induced by the *Plasmodium* infection.

**IMPORTANCE:**

Malaria-associated lung pathology is a common complication of malaria in adults and often occurs during or even after antimalarial treatment, and current evidence suggests that it is associated with cytokine imbalance and dysregulation of immune responses in the lungs. In this study, we conducted detailed flow cytometry analyses, time-series bulk transcriptomics, and spatial transcriptomics to profile the immune landscape of malaria-associated lung pathology in a mouse malaria model and revealed that IFN-γ signaling in T cells plays a key role in the lung pathology. In addition, we identified a subgroup of CD8-expressing proinflammatory monocytes that exhibit heightened parasite phagocytotic capability.

## INTRODUCTION

Malaria caused a severe global health burden by causing almost 282 million estimated malaria cases in 2024, with the African Region carrying both the heaviest burden of the disease and mortality (1). The clinical presentation of severe malaria varied (2), including cerebral and pulmonary manifestations. Lung pathology was reported in cases of many *Plasmodium* species, including *P. falciparum*, *P. vivax,* and *P. knowlesi* (3).

Severe malaria with lung involvement is determined as confirmed edema on radiologic examination or an oxygen saturation of lower than 92%, while breathing ambient air, with a respiratory rate of more than 30 breaths per minute (2). The lethal form of malaria-associated (MA) lung pathology, including acute lung injury and acute respiratory distress syndrome (ARDS), typically occurs in adults (3) and is considered to be caused by the parasite-induced systemic inflammation, even after antimalarial treatment (4–7). It was reported that treatment with the anti-inflammatory glucocorticoid dexamethasone can alleviate lung injury and prolong the survival of mice, suggesting that inflammation is one of the major contributors to MA-ARDS (8).

Although the initial trigger of inflammation has not been clearly understood, leukocyte accumulation, adhesion of infected red blood cells (iRBCs), and the release of inflammatory compounds are thought to be the main contributors to lung pathology (9–13). Endothelial activation and pulmonary injury occur after sequestration of iRBCs and leukocytes (9). Inflammatory cells, such as neutrophils, are recruited by the proinflammatory mediators and accumulate in the lung alveoli and microcirculation, leading to further insult to the alveolar epithelium and vascular endothelium (14, 15). As one of the major types of leukocytes infiltrated in the lungs during malaria infection, neutrophils facilitate defense responses via reactive oxygen species production, phagocytosis, neutrophil extracellular traps (NETs), secretion of cytokines and chemokines, and antigen presentation (16, 17). However, NETs can also induce lung injury by directly killing epithelial and endothelial cells and impairing the autophagic flux in alveolar epithelial cells (18–20), and treatment with NET inhibitors was reported to attenuate lung pathology (21). Post-mortem analysis of deceased MA-ARDS patients revealed the accumulation of other types of leukocytes, including monocytes, macrophages, and T cells (8, 22). Single-cell RNA sequencing (scRNA-seq) analysis also showed dramatic increase in many immune cell types in a mouse model of MA-ARDS, including proliferating T cells, T effector cells, cytotoxic T effector cells, natural killer (NK) cells, neutrophils and macrophages (23).

In this study, we utilized a mouse malaria model, *P. berghei* NK65-infected C57BL/6J mice, to analyze the immune responses in malaria-associated lung pathology. Utilizing time-series bulk transcriptomics and spatial transcriptomics analyses, we found that upregulated IFN-γ signaling in T cells contributes to lung pathology. Disruption of IFN-γ signaling in T cells alleviated malaria-associated lung pathology, and this attenuation of pathology is associated with an enhanced T cell-monocyte interaction and an increased population of monocytes with heightened phagocytic capability, which express CD8 and Ly6C.

## RESULTS

### Malaria-associated lung pathology is associated with the accumulation and activation of T cells and monocy*tes*

**Fig 1:**
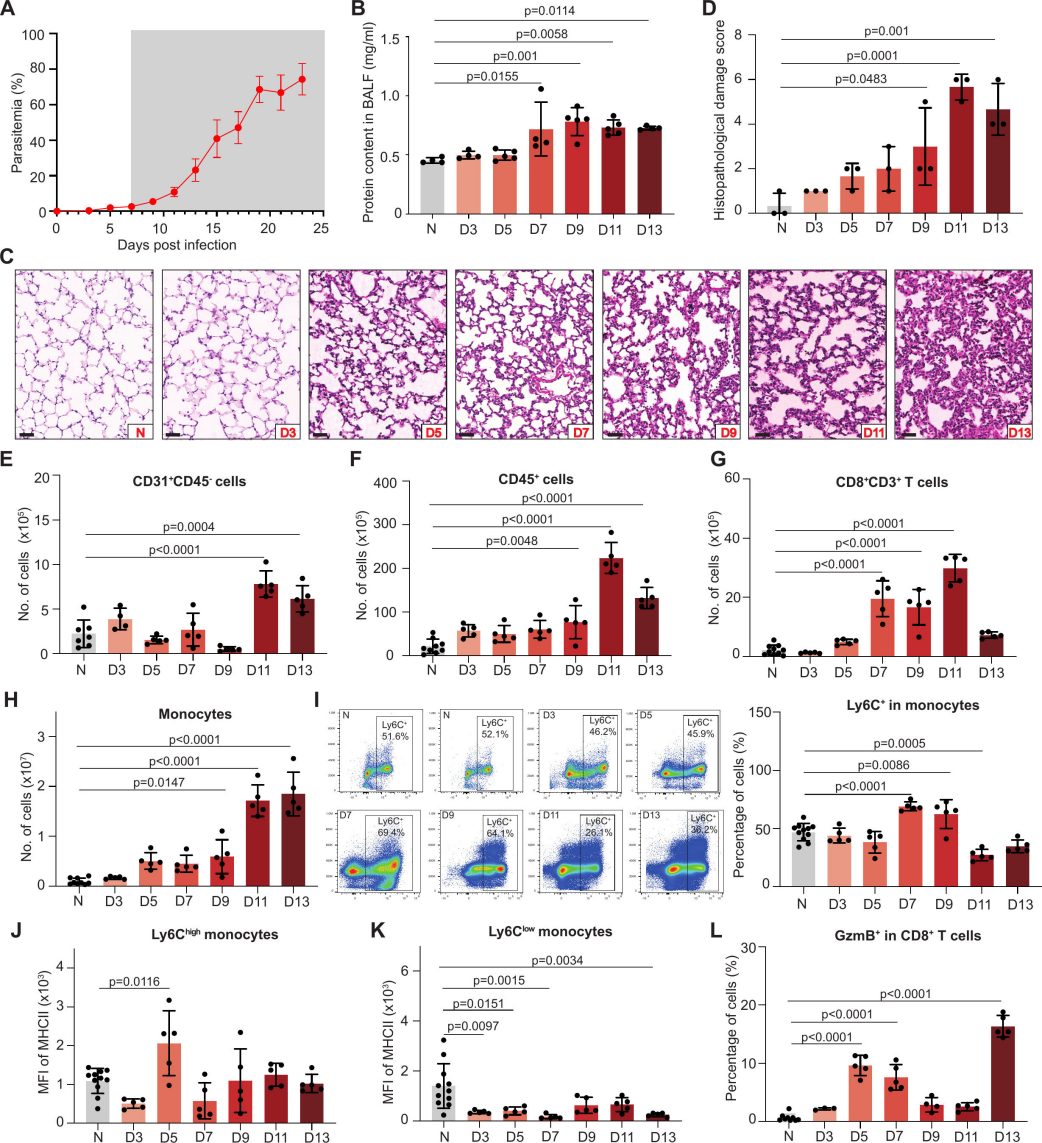
The pathology and immune landscape in the lungs of mice developed malaria-associated lung pathology. (**A**) Parasitemias of C57BL/6J mice (*n* = 6) infected with *P. berghei* NK65. The time points exhibiting lung pathology are shaded in gray. Means with SD are shown. (**B**) Protein content in the BALF of naïve or infected mice. N, naïve; D, days post-infection. Each dot represents an individual mouse (*n* = 4–5). The data are representative of two independent experiments. Error bars, SD; *P*-values, one-way ANOVA with a *post hoc* Tukey’s HSD test in panels **B, D, E–L**. (**C**) Representative photomicrographs of hematoxylin and eosin (H&E)-stained lung sections of naïve and infected mice (*n* = 3). Scale bar, 50 μm. (**D**) Histopathological damage score derived from H&E-stained lung sections (*n* = 3). The cell numbers of CD31^+^CD45^−^ cells (**E**) and CD45^+^ cells (**F**) in the lungs of naïve or infected mice (*n* = 5–9). Naïve samples were collected at different time points together with the infected samples to ensure the accuracy of the analysis. The cell numbers of CD8^+^CD3^+^ T cells (**G**) and CD11b^+^SiglecF^−^Ly6G^−^CD11^-−^ monocytes (**H**) in the lungs of naïve or infected mice (*n* = 5–10). (**I**) The expression level of Ly6C in the monocytes. Left panel, representative flow cytometry plots; right panel, the percentage of Ly6C^+^ monocytes at different time points (*n* = 5–11). Mean fluorescence intensity (MFI) of MHC II on Ly6C^high^ monocytes (**J**) and Ly6C^low^ monocytes (**K**) (*n* = 5–11). (**L**) The percentages of CD8^+^ T cells expressing GzmB (*n* = 3–9).

*P. berghei* NK65 (PbNK65) infected C57BL/6J mice (with a dose of 10^4^ iRBCs) showed signs of hyperventilation at 7 days post-infection (dpi), with a mean parasitemia of 2.65%. The symptoms of lung pathology clearly developed at 9 dpi and persisted until the mice died from hyperparasitemia (parasitemia above 85%) and emaciation (weight loss of more than 25%) (Fig. 1A; Fig. S1A and B*).* The protein content in bronchoalveolar lavage fluid (BALF) increased by 58% and 72% at 7 and 9 dpi, respectively (Fig. 1B). Vascular permeability, as evidenced by Evans blue leakage in the lungs, was observed at 9 dpi (Fig. S1C), in line with previous studies (23–25)*.*

As early as 3 dpi, inflammatory immune cells, mainly plasma cells and monocytes, were observed at the alveolar walls and alveoli, and the accumulation of immune cells increased as the infection advanced (Fig. 1C). Alveolar walls thickened slightly at 5 dpi, and the thickness increased as the infection progressed. Alveolar edema was noticeable at 9 dpi, and the alveolar area decreased dramatically at 13 dpi. In addition, hemozoins were observed and distributed at the alveolar walls and pleura during the infection (Fig. 1C). The histopathological damage was pronounced since 9 dpi and continuously worsened until 13 dpi (Fig. 1D).

To further profile the immune cells, we conducted flow cytometry analysis on the lungs of naïve and infected mice (Fig. 1E-L; Fig. S1D-K; Fig. S2; antibody panel was summarized in Table S1). The numbers of CD31^+^ endothelial cells and CD45^+^ leukocytes peaked at 11 dpi (Fig. 1E and F; Fig. S1F and G). The major immune cell types that accumulated in the lungs of infected mice were CD8^+^ T cells and monocytes, as their cell numbers both increased by more than twice compared to the naïve mice at 5 dpi and continuously increased to 13.7 times and 18.8 times at later infection, respectively (Fig. 1G and H; Fig. S1I and S2B).

The percentage of proinflammatory Ly6C^+^ monocytes elevated by 47% and 33% at 7 and 9 dpi, respectively (Fig. 1I; Fig. S2C), and the level of MHC II on Ly6C^high^ monocytes was nearly doubled at 5 dpi compared to the naïve mice (Fig. 1J; Fig. S2D). In sharp contrast, the MHC II level significantly decreased on Ly6C^low^ monocytes upon infection (Fig. 1K; Fig. S2E). The percentage of CD8^+^ T cells expressing Granzyme B (GzmB) sharply increased by 10 times at 5–7 dpi compared to that of naïve mice (Fig. 1L).

Taken together, PbNK65 infection impaired the integrity of the lung vessels, induced airspace flooding and inflammation typified by the accumulation and activation of cytotoxic T cells and monocytes.

### Time-series transcriptomics analysis of the lungs of infected mice revealed an elevated T cell response at the onset of malaria-associated lung pathology

**Fig 2:**
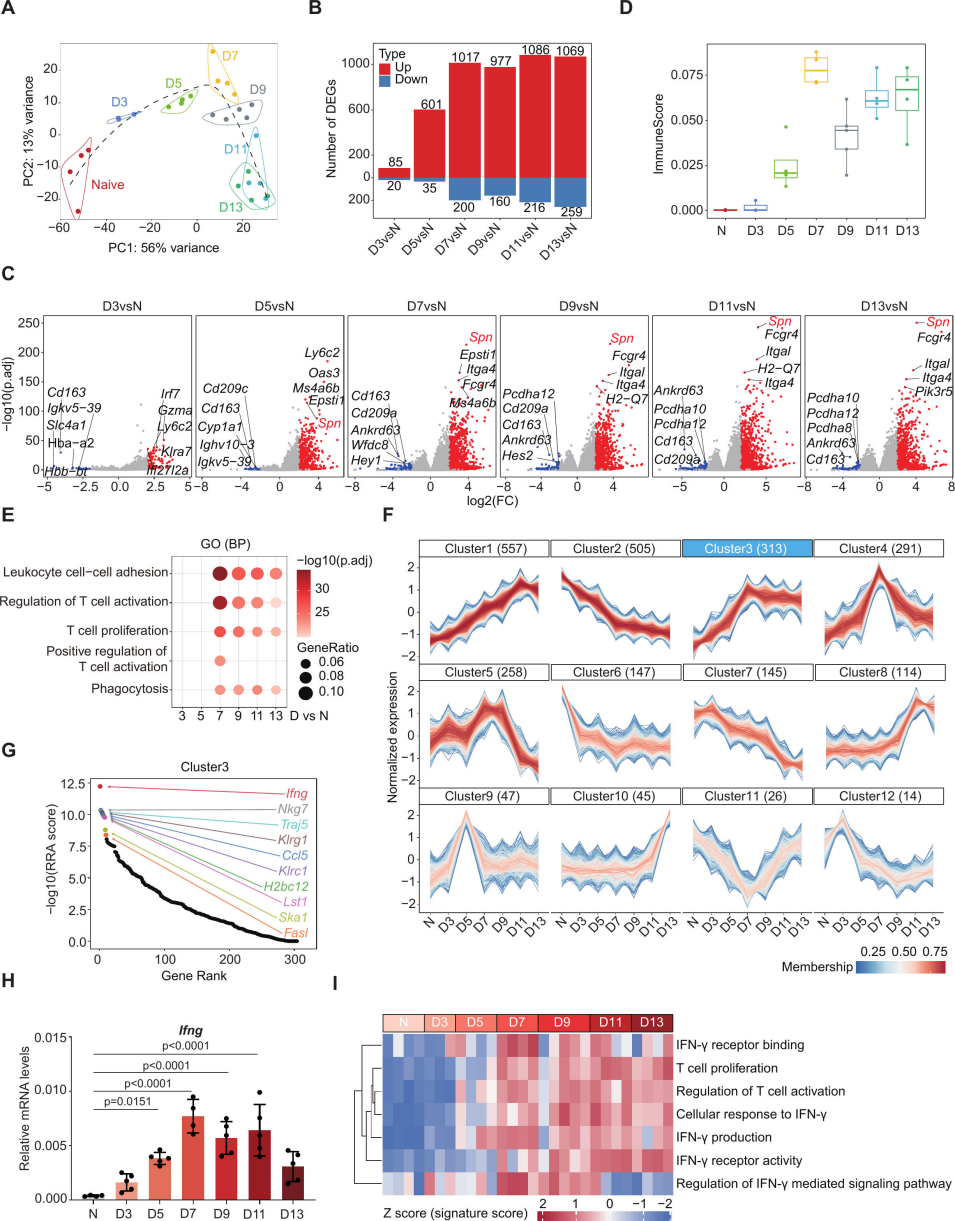
Time-series transcriptomics analysis of the lungs of PbNK65-infected mice. (**A**) PCA of the whole transcriptomes of lungs from naïve (N) and infected mice at different days post-infection (D). Each dot represents an individual mouse, and the samples are colored by the infection status (*n* = 4). The dashed lines indicate the trajectories of the samples progressing over time, with D7 marking a turning point. (**B**) The numbers of DEGs identified by DESeq2 [|log2 (fold change)| > 2 and adjusted *P*-value < 0.05, Benjamini-Hochberg (BH) correction] between the infected and naïve lungs. The red bars represent the numbers of upregulated genes, while the blue bars represent the numbers of downregulated genes. The numbers of DEGs are provided above or below each bar. (**C**) The volcano plots of the DEGs shown in panel **B** with the top 5 upregulated (red) and downregulated (blue) genes highlighted, sorted by the adjusted *P*-values. (**D**) The box plot showing the ImmuneScore of the whole lung transcriptomes shown in panel **A**, indicating the level of immune cell infiltration. (**E**) GO enrichment analysis showing the biological processes (BP) upregulated at 7 dpi. The sizes of the symbols indicate the ratio of genes enriched in this pathway and the color scale indicates −log10(adjusted *P*-values) with BH correction. (**F**) Gene clusters based on the expression profiles of the genes. The color scale represents the membership value, and the numbers in the brackets indicate the number of genes in each cluster with a membership value ≥0.75. (**G**) Dot plot displays the robust rank aggregation (RRA) analysis of the genes in Cluster 3 highlighted in panel **F**, genes with the top 10 RRA scores are shown and highlighted. (**H**) Relative mRNA level of *IFNG* in the lungs of naïve or infected mice (*n* = 4–5). Mouse β-actin was used as an internal control. Each dot represents an individual mouse. Error bars, SD. *P*-values, one-way ANOVA with a *post hoc* Tukey’s HSD test. (**I**) Heatmap of the signature scores of the indicated pathways derived from gene set variation analysis (GSVA). Color scale, Z score of the signature scores.

To investigate the immune responses during the manifestation of the lung pathology, we conducted a time-series transcriptomics analysis on a total of 28 lung samples collected at six time points: 3, 5, 7, 9, 11, and 13 dpi, along with the lungs of naïve mice as controls (four mice per group). The principal component analysis (PCA) revealed that the transcriptomes of the lungs were significantly altered as early as 3 dpi, with the infected samples diverging from the naïve samples on PC1 (Fig. 2A). Interestingly, the transcriptome at 7 dpi differed the most from the naïve samples on PC2 (Fig. 2A). The hierarchical clustering based on Euclidean distance showed that the samples were clearly separated into two clusters, one including naïve samples and samples collected before 7 dpi, and the other consisting of samples from 7 to 13 dpi (Fig. S3A). The number of differentially expressed genes (DEGs; |log2 (fold change)| > 2, adjusted *P*-value < 0.05) compared to naïve lungs steeply increased at 5 and 7 dpi, with the number of downregulated genes showing a sharp increase at 7 dpi (Fig. 2B; Table S3). Notably, *Spn*, a gene involved in antigen-specific activation of T cells, was upregulated and maintained as the top upregulated gene since 7 dpi (Fig. 2C). The ImmuneScore (indicating the level of immune cell infiltration) peaked at 7 dpi (Fig. 2D). We next performed Gene Ontology (GO) analysis on DEGs at each time point (Table S4), and the analysis showed the enrichment of “Leukocyte cell-cell adhesion,” “Regulation of T cell activation,” and “T cell proliferation” as early as 7 dpi, with the adjusted *P*-values gradually declining at the later infection (Fig. 2E). Altogether, these results indicated that 7 dpi may be a “decisive” time point for the development of malaria-associated lung pathology.

We further explored the dynamic transcriptomic changes throughout the course of infection by clustering the genes based on their expression profiles using Mfuzz (26) resulting in 12 distinct clusters (Fig. 2F; Table S5). GO analysis revealed that genes from Clusters 1, 3, 4, 6, and 9 were associated with immune-related pathways (Fig. S3B; Table S5). Among these, Cluster 3 resembled the profile of ImmuneScore the most, showing gene expression gradually increasing after the infection and peaking at 7 dpi (Fig. 2F). Notably, the genes in Cluster 3 were enriched with “Lymphocyte proliferation,” “T cell receptor signaling pathway,” and “Regulation of T cell activation” (Fig. S3B). We used the RRA analysis (27) to rank the DEGs in Cluster 3 and found that *Ifng* ranked at the top (Fig. 2G). The transcription of *Ifng* was highly upregulated at 7 dpi and remained highly expressed at 9–11 dpi (Fig. S3C), which was confirmed by qRT-PCR analysis (Fig. 2H). Consistently, gene set variation analysis (GSVA) showed that the pathways such as “IFN-γ production,” “IFN-γ receptor binding,” and “T cell proliferation” were upregulated at 7 dpi and maintained at high levels onwards (Fig. 2I). Gene set enrichment analysis (GSEA) also revealed that these pathways were significantly enriched at 7 dpi (Fig. S3D through G).

Taken together, these data suggest that PbNK65 infection triggered a strong immune response in the lungs during infection, and IFN-γ response in T cells may be important in malaria-associated lung pathology.

### IFN-γ signaling in T cells contributes to the development of malaria-associated lung pathology

**Fig 3:**
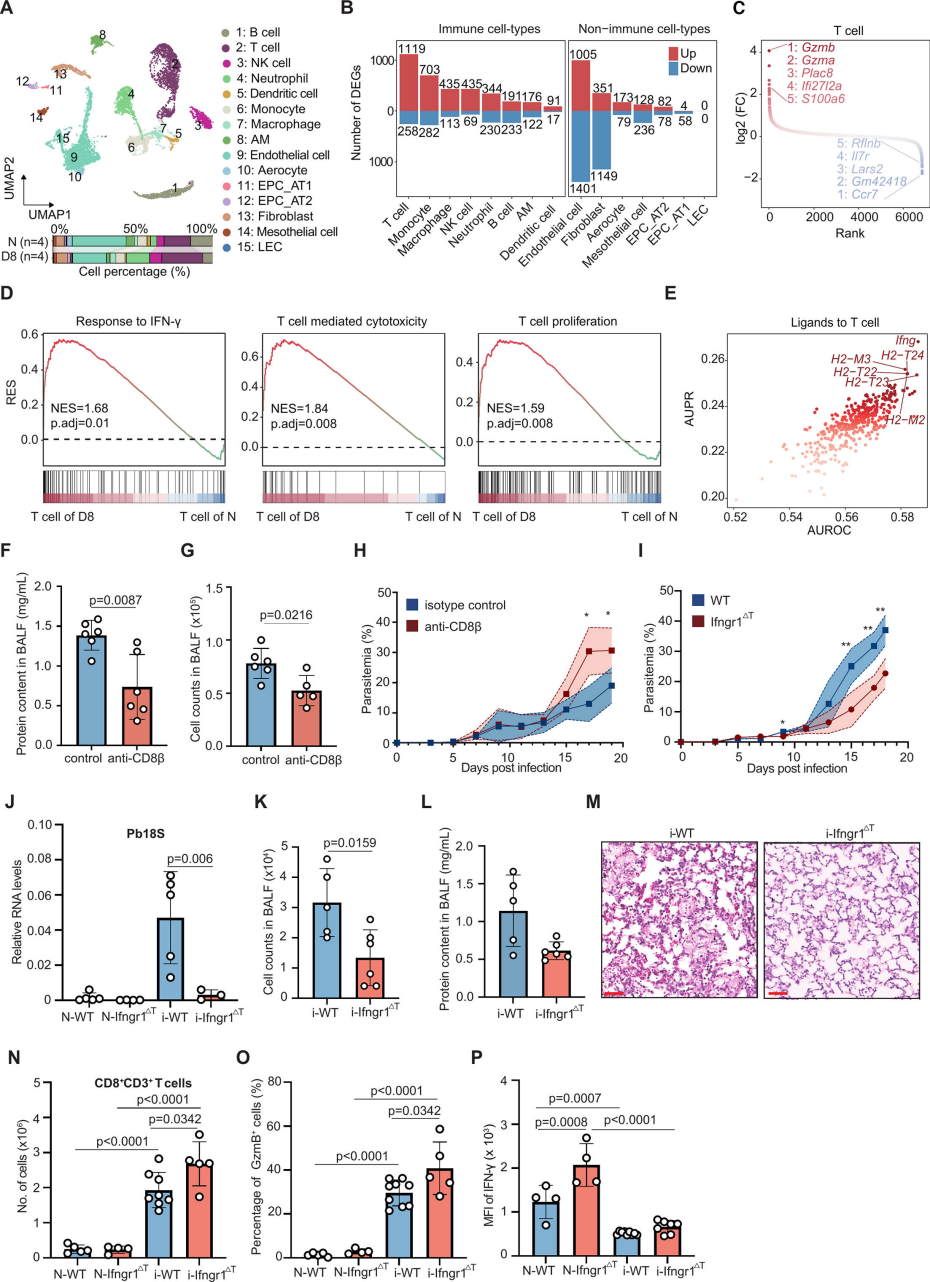
IFN-γ signaling in T cells contributes to malaria-associated lung pathology. (**A**) UMAP of scRNA-seq data of a mouse malaria-associated ARDS model. A bar chart in the lower panel displays the distribution of the cell types in naïve (N) and infected (D8) samples. AM, alveolar macrophage; EPC_AT1/2, type 1/2 alveolar epithelial cell; LEC, lymphatic endothelial cell. (**B**) The number of DEGs with |log2 (fold change)| > 0.25 and adjusted *P*-value <0.05, determined by Wilcoxon rank-sum test with Bonferroni correction. Red bars indicate the numbers of genes upregulated in the infected lungs, and blue bars represent downregulated genes. (**C**) Dot plot showing the DEGs in T cells, with the top 5 up- or downregulated genes highlighted. The genes were ranked by log2 (fold change). (**D**) GSEA of DEGs in T cells between infected and naïve groups, highlighting the pathways related to IFN-γ and T cell responses. (**E**) Ligands signaling to T cells, with the *x*-axis representing the area under the receiver operating characteristic curve (AUROC) and the *y*-axis representing the area under the precision-recall curve (AUPR). The top 5 ligands were highlighted. The color intensity represents the ranking of the ligands, with the darker color indicating the higher ranking. Total protein content (**F**) and cell count (**G**) in the BALF of 10^4^ PbNK65-infected mice (*n* = 5–6) treated with anti-CD8β or isotype control antibodies. Each dot represents an individual mouse. *P*-value, Mann-Whitney U-test. Mice were intravenously injected with 50 μg anti-CD8β antibodies or antibody isotype controls 1 day before intraperitoneal (i.p.) injection of 10^4^ PbNK65-iRBCs. An additional 20 μg antibody injection was performed at 4 dpi, and the BALF was collected at 8 dpi. (**H**) Parasitemias of infected mice treated with anti-CD8β or isotype control antibodies (*n* = 6). The mice received three injections of antibodies 1 day before PbNK65 infection and 4 and 8 dpi. (**I**) Parasitemias T cell-specific IFN-γR1 knockout mice (*Ifngr1*^ΔT^, *Ifngr1* ^flox/flox^ lck-icre^+/−^) compared to wild-type controls (WT, lck-icre^+/−^) infected with 10^4^ PbNK65-iRBCs. The results were representative of three independent experiments (*n* = 5–9). The box symbols represent the means and SD were shown as the light shadow of red and blue. *, *P*-values < 0.05; **, *P*-values < 0.01; Mann-Whitney U-test. (**J**) Relative parasite 18S level in the lungs of naïve (N-) or 10^4^ PbNK65-iRBCs infected (i-) WT or *Ifngr1*^ΔT^ mice at 8 dpi (*n* = 3–5). The lungs were collected after extensive transcardiac perfusion. Mouse β-actin was used as an internal control. Each dot represents an individual mouse. Error bars, SD; *P*-values, one-way ANOVA with a *post hoc* Tukey’s HSD test in panels **J and N–P**. Total cell counts (**K**) and protein content (**L**) in the BALF of 10^4^ PbNK65-iRBCs infected mice collected at 8 dpi. Each dot represents an individual mouse. Error bars, SD. *P*-values, Mann-Whitney U-test. The results were representative of two independent experiments (*n* = 5–6). (**M**) Representative H&E-stained sections of the lungs collected from mice shown in panels **K and L**. Scale bar, 50 μm. (**N**) The cell number of CD8^+^CD3^+^ T cells in the lungs of uninfected and 10^4^ PbNK65-iRBCs infected mice at 8 dpi (*n* = 4–8). (**O**) The proportion of GzmB-expressing CD8^+^CD3^+^ T cells in the lungs of naïve and PbNK65-infected mice at 8 dpi. Each dot represents an individual mouse (*n* = 4–9). (**P**) MFI of IFN-γ level in CD8^+^CD3^+^ T cells (*n* = 4–9). The cells were stimulated by phorbol 12-myristate 13-acetate (PMA) and ionomycin with the presence of GolgiStop before intracellular staining.

Given that the time-series transcriptomics analysis indicated T cell activation is important for the development of malaria-associated lung pathology, we next sought to examine whether the MA-ARDS showed similar immune responses. We re-analyzed a published scRNA-seq data set of a murine model of MA-ARDS (23), which included a total of 10,545 single-cell transcriptomes from four naïve and four PbNK65-infected lung samples at 8 dpi (Fig. 3A; Fig. S4A and B). Similar to our milder lung pathology model, the proportions of T cells and neutrophils in MA-ARDS were elevated, and the fractions of endothelial cells, dendritic cells, and alveolar macrophages were reduced (Fig. S4C and D).

We performed the DEG analysis for the 15 identified cell types and found that T cells exhibited the most pronounced transcriptional changes after infection, with 1,119 upregulated genes and 258 downregulated genes [|log2 (fold change)| > 0.25, adjusted *P*-value < 0.05] (Fig. 3B; Table S6). The top upregulated genes were primarily associated with cytotoxicity, including *Gzmb* (upregulated by 17-fold) and *Gzma* (upregulated by 10-fold) (Fig. 3C). GSEA indicated that the pathways of “Response to IFN-γ,” “T cell-mediated cytotoxicity,” and “T cell proliferation” were significantly activated in T cells of the infected lungs (Fig. 3D), consistent with the time-series transcriptomics data. Furthermore, we examined cell communication signals of T cells (as recipients) using NicheNet (28), and identified IFN-γ as the top ligand (Fig. 3E).

It was reported that CD8^+^ T cells are the main IFN-γ producers in PbNK65 infection model (25); we therefore treated the PbNK65-infected mice with anti-CD8β or isotype control antibodies and analyzed the lung pathologies. Both the protein content and cell counts in the BALF of the infected mice treated with anti-CD8β were significantly downregulated compared to the mice treated with isotype control at 8 dpi (Fig. 3F and G). The parasite sequestration level (quantified as 18S of *P. berghei* NK65 relative to mouse β-actin) did not show significant difference despite a trend of increase observed in anti-CD8β treated mice (Fig. S6A). The parasitemia did not show a difference until 17 dpi when the parasitemia was 2.3 times higher in anti-CD8β treated mice compared to the control (Fig. 3H). To further analyze the T cell responses, we utilized T cell-specific *Gzmb* (*Gzmb*^ΔT^, *Gzmb*^flox/flox^ lck-iCre^+/−^, Fig. S5B) and *Ifngr1* knockout mice (*Ifngr1*^ΔT^, *Ifngr1*^flox/flox^ lck-iCre^+/−^, Fig. S5C through F). The age-matched (8–12 weeks) conditional knockout mice and wild-type controls (lck-iCre^+/−^, WT) were infected with 10^4^ PbNK65-iRBCs. Interestingly, both the parasitemias and pathology did not show differences in the infected *Gzmb*^ΔT^ mice compared to the infected WT mice (Fig. S6B through D), while the parasitemias of infected *Ifngr1*^ΔT^ mice showed a significant decrease at 9 dpi and a 57% reduction at 15 dpi compared to the control (Fig. 3I).

A previous study showed that different doses of iRBCs may trigger differential IFN-γ responses (29). With a higher dose of infection (10^6^ *P. berghei* ANKA-iRBCs) in 129 SV/EV mouse background, the *Ifngr1* knockout mice developed higher parasitemia than the WT mice as early as 7 dpi. In contrast, no differences in parasitemia were observed with a lower dose of infection (5 × 10^5^ *P. berghei* ANKA-iRBCs) before WT mice developed cerebral pathology (29). We therefore also infected the *Ifngr1*^ΔT^ and WT mice with a high dose of 10^6^ PbNK65-iRBCs and analyzed the parasitemia. Different from the dose of 10^4^ iRBCs, the high dose of infection induced an early peak of infection at 5 dpi in *Ifngr1*^ΔT^ mice; however, the parasitemias were not significantly different (Fig. S6E). We therefore used the infection dose of 10^4^ PbNK65-iRBCs in later experiments to analyze the lung pathology in *Ifngr1*^ΔT^ mice.

We analyzed the parasite loads in the lungs of infected mice at 8 dpi and found the level of parasite 18S mRNA decreased by 93% in the lungs of infected *Ifngr1*^ΔT^ mice compared to the WT controls (Fig. 3J). Importantly, the symptoms of lung pathology in the infected *Ifngr1*^ΔT^ mice were attenuated, as the mice exhibited slower and easier breathing compared to the infected WT mice. A 58% decline in the accumulated cells in the BALF was observed in the infected *Ifngr1*^ΔT^ mice compared to the WT mice at 8 dpi (Fig. 3K), and the protein content also showed a trend of decrease at 8 dpi (Fig. 3L). Moreover, the alveolar epithelium damage was decreased (Fig. 3M). The numbers of total lung cells and CD45^+^ leukocytes were comparable in the uninfected mice, whereas the counts were significantly decreased in the infected *Ifngr1*^ΔT^ mice compared to infected WT mice at 8 dpi (Fig. S6F and G). The cell numbers of alveolar macrophages, Ly6C^-^ monocytes, and Ly6C^+^ monocytes showed no differences between the uninfected groups, but all showed a sharp decrease in the infected *Ifngr1*^ΔT^ mice compared to the WT controls at 8 dpi (Fig. S6H through J). In contrast, the cell numbers of DCs and neutrophils showed no significant changes in the lungs of both uninfected and infected *Ifngr1*^ΔT^ mice (Fig. S6K and L). The biggest changes lay in CD8^+^ T cells, as although the cell number of CD8^+^ T cells did not differ between the uninfected *Ifngr1*^ΔT^ and WT mice, the numbers increased by 38% in the infected *Ifngr1*^ΔT^ mice compared to the WT mice (Fig. 3N; Fig. S6M). In addition, the fraction of GzmB-expressing cells was elevated in CD8^+^ T cells of the infected *Ifngr1*^ΔT^ mice (Fig. 3O), indicating that the CD8^+^ T cells in the infected *Ifngr1*^ΔT^ mice were more activated compared to the WT controls. Interestingly, the level of IFN-γ (MFI) after PMA and ionomycin stimulation was decreased in IFN-γ^+^CD8^+^ T cells after infection in both *Ifngr1*^ΔT^ and WT mice (Fig. 3P; Fig. S6N).

In short, the loss of *Ifngr1* in T cells, but not *Gzmb* in T cells, attenuated malaria-associated lung pathology. CD8^+^ T cells may play an important role in the lung pathology, and they were more activated in the infected *Ifngr1*^ΔT^ mice.

### Spatial transcriptomics analysis revealed enhanced T cell-monocyte interaction in the infected *Ifngr1*^ΔT^ mice

**Fig 4:**
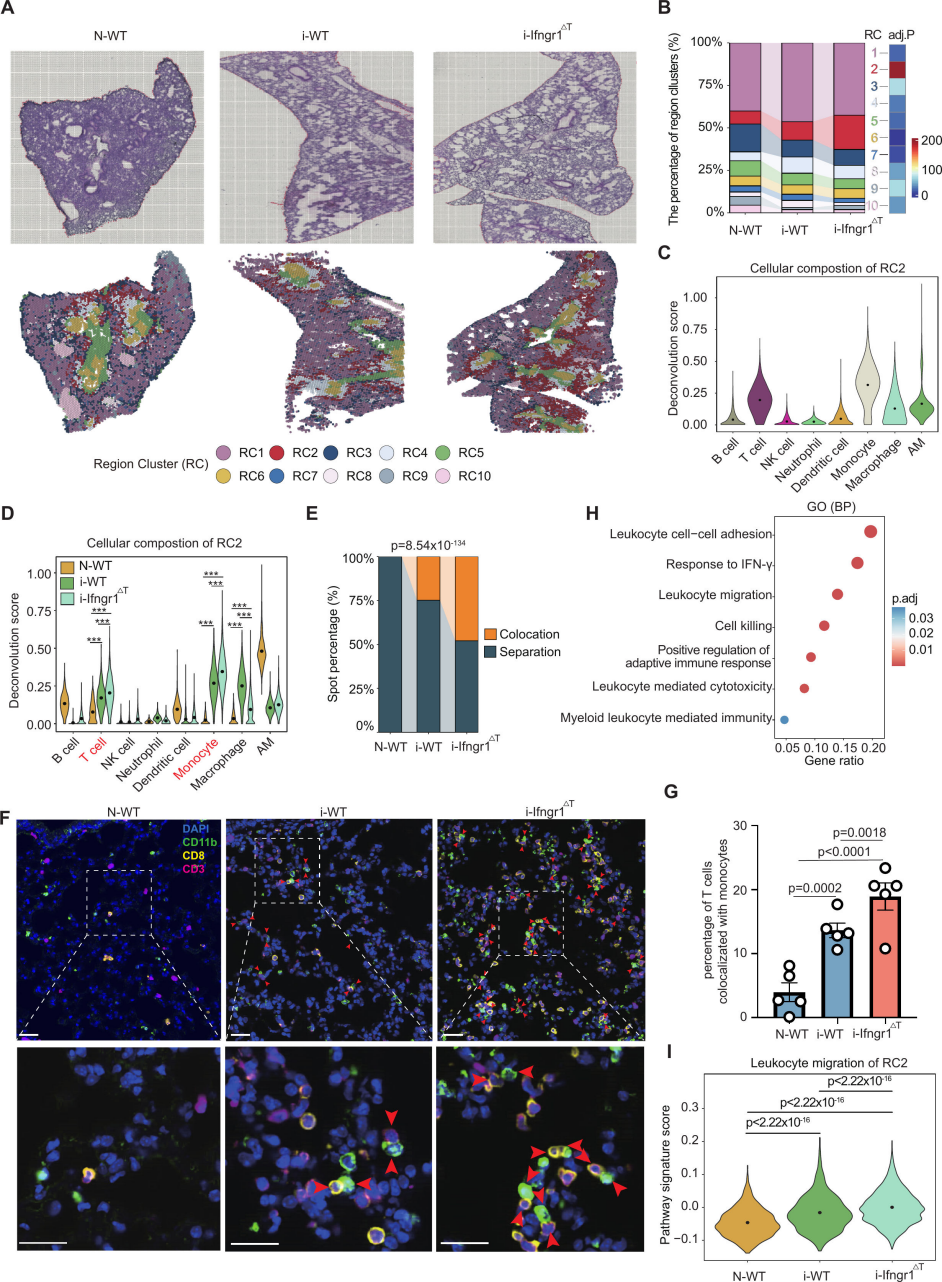
Spatial transcriptomic analysis revealed enhanced T cell-monocyte interaction (**A**) Spatial transcriptomic (ST) analysis identified region clusters altered after infection. Upper panel, H&E-stained sections of lungs isolated from naïve wild-type (N-WT) mouse and wild-type (i-WT) or Ifngr1^ΔT^ (i-Ifngr1^ΔT^) mouse infected with 10^4^ PbNK65-iRBCs. The infected lungs were isolated at 7 dpi. Lower panel, ST spots colored by identified RC. (**B**) Stacked bar chart illustrating the proportions of region clusters in the lungs of N-WT, i-WT, and i-Ifngr1^ΔT^ mice, ranked by the percentages in the naïve WT mouse. The bar chart on the right represents the −log10(adjusted *P*-value) for each RC, derived from a χ^2^ test corrected using the Holm method. Color scale, −log10(adjusted *P*-value). (**C**) Deconvolution of the immune cells in RC2, shown as violin plot colored by the immune cell types. AM, alveolar macrophage. Black dots represent the mean values. (**D**) Deconvolution of immune cells in RC2 of N-WT, i-WT, and i-Ifngr1^ΔT^ lungs, shown as violin plot colored by the samples. *** indicates adjusted *P*-values <0.001, multiple *t*-test corrected by the Holm method. (**E**) Percentages of spots with T cells and monocytes colocalized or separated (spots containing only monocytes or T cells) in RC2 of N-WT, i-WT, and i-Ifngr1^ΔT^ lungs. *P*-value, χ^2^ test. (**F**) Immunofluorescence analysis showing the co-localization of monocytes and T cells in the lungs of N-WT, i-WT, and i-Ifngr1^ΔT^ mice (*n* = 5). The slides were stained with anti-CD11b (green), anti-CD3 (magenta), and anti-CD8 (yellow) antibodies and DAPI (blue). Red arrows indicate the colocalization of (CD8^+^) CD3^+^ T cells and monocytes. Scale bar, 20 μm. (**G**) The percentage of T cells (CD3^+^) colocalized with monocytes (CD11b^+^) in 20 FOVs (282 × 176 μm^2^, avoiding big air tubes and blood vessels) randomly selected in the lung sections of naïve WT mice or infected mice at 8 dpi (*n* = 5). Error bars, SD; *P*-values, one-way ANOVA with a *post hoc* Tukey’s HSD test. (**H**) GO enrichment analysis for DEGs [log2 (fold change) > 0 and adjusted *P*-value <0.05] in RC2 compared to the rest of the region clusters. BP, biological pathway. Gene ratio indicates the ratio of DEGs enriched in this pathway. Color scale represents the adjusted *P*-values. (**I**) Violin plot showing the signature scores of the pathway of leukocyte migration in RC2 of N-WT, i-WT, and i-Ifngr1^ΔT^ lungs. *P*-values, Wilcoxon rank-sum test.

To further analyze the interaction between T cells and the innate cells, we performed a spatial transcriptomics on the lungs isolated from *Ifngr1*^ΔT^ and WT mice infected with 10^4^ PbNK65-iRBCs at 7 dpi, along with a naïve mouse. After stringent quality control, we obtained transcriptomes from 38,909 high-quality spots clustered into 10 distinct region clusters (RC) by unsupervised clustering (Fig. 4A; Fig. S7A and B). Notably, a significant increase (adjusted *P*-value = 9.13 × 10^−195^, Holm-adjusted χ^2^ test) of RC2 was observed in the infected WT mouse lung (10.8%) compared to the naïve (7.6%), and the increase was further enlarged to 20.1% in infected *Ifngr1*^ΔT^ mouse lung (Fig. 4B). RC2 represents regions surrounding the tracheal epithelium, which includes tissues adjacent to the tracheal lining and contains immune cells and structural elements supporting the epithelium. We performed a cell deconvolution analysis and found that RC2 predominantly consisted of monocytes and T cells (Fig. 4C). The deconvolution scores of two cell types were sharply increased after infection: T cells increased by 2-fold, and monocytes increased by 11-fold. The fractions of the two cell types were even higher in the lung section of infected *Ifngr1*^ΔT^ mouse compared to the infected WT mouse (Fig. 4D). Importantly, the percentage of T cell-monocyte colocalization likely increased by 23% in the infected *Ifngr1*^ΔT^ mouse compared to WT mouse (Fig. 4E). Immunofluorescence analysis confirmed that both the cell numbers and the colocalization/interaction between monocytes (CD11b^+^) and CD8^+^ T cells (CD8^+^CD3^+^) were increased after infection, and the increase was more pronounced in the infected *Ifngr1*^ΔT^ mice (Fig. 4F). We quantified the rate of T cell-monocyte colocalization and found a 38% increase of colocalization in the lungs of infected *Ifngr1*^ΔT^ mice compared to the infected WT mice (Fig. 4G; Fig. S7C), particularly the rate of CD8^+^ T cell-monocyte colocalization doubled in the infected *Ifngr1*^ΔT^ mice compared to the infected WT mice (Fig. S7D and E).

Correspondingly, the differential expression analysis [log2 (fold change) > 0, adjusted *P*-value < 0.05] (Table S7) revealed that genes specific to RC2 were enriched in the pathways related to “response to IFN-γ,” “leukocyte cell-cell adhesion,” and “leukocyte migration” (Fig. 4H; Fig. S7F; Table S7). The pathway signature score of “leukocyte migration” was significantly upregulated in the infected WT mouse compared to the naïve WT mouse, and the score was further increased in the infected *Ifngr1*^ΔT^ mouse (Fig. 4I).

Taken together, T cells and monocytes were clustered in the regions surrounding the tracheal epithelium after infection, and IFN-γ signaling in T cells likely mediates leukocyte migration and T cell-monocyte interaction.

### CD8^+^Ly6C^+^ monocytes increased in the *Ifngr1*^ΔT^ mice exhibiting enhanced parasite phagocytic capability

**Fig 5:**
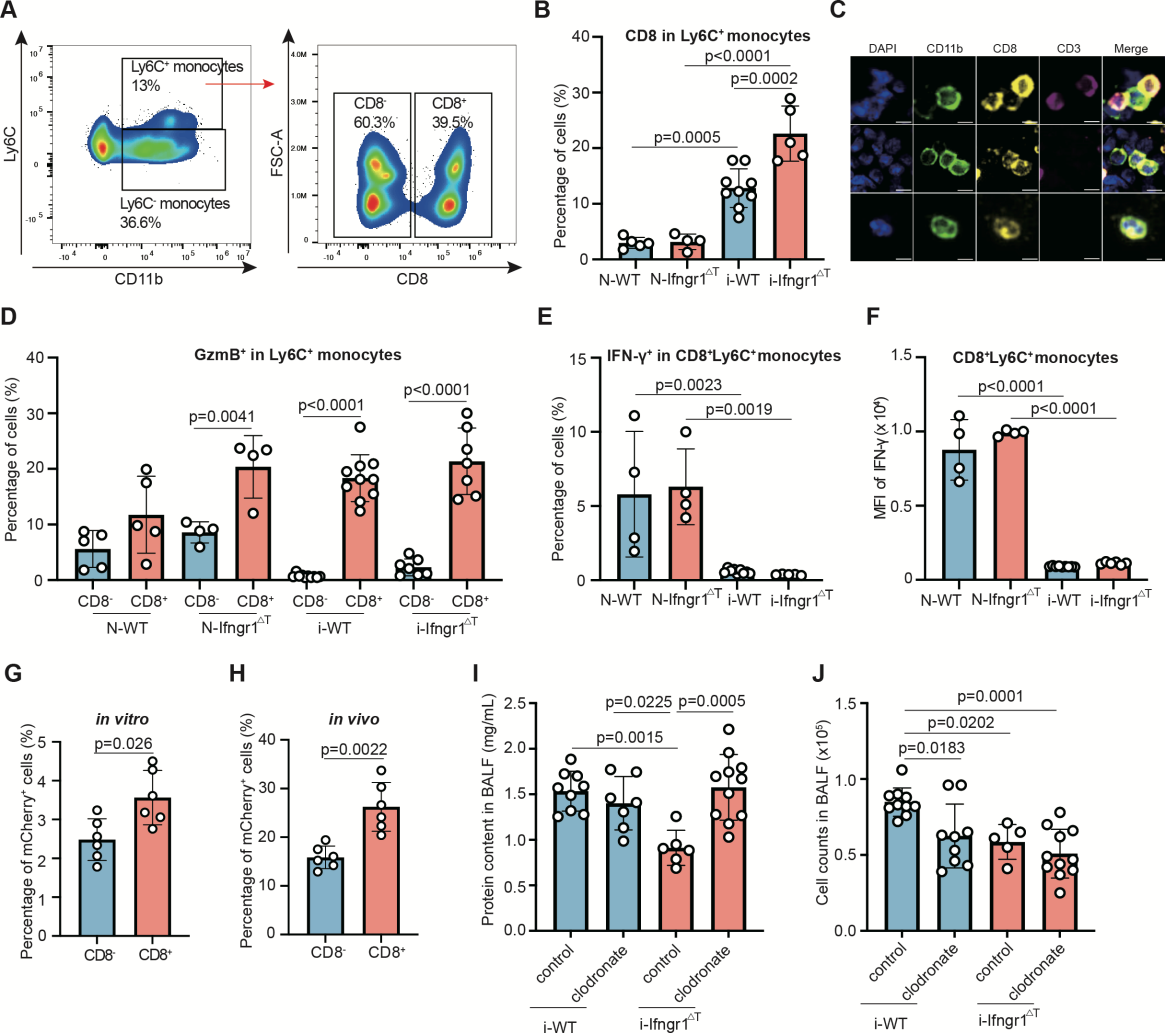
CD8^+^Ly6C^+^ monocytes increased after infection and displayed enhanced phagocytic activity. (**A**) Representative flow cytometry dot plot showing CD8 expression on CD11b^+^Ly6C^+^ monocytes. (**B**) The percentages of Ly6C^+^ monocytes expressing CD8 in the lungs of naïve wild-type (N-WT) mice, and lungs of 10^4^ PbNK65-infected wild-type (i-WT) or Ifngr1^ΔT^ (i-Ifngr1^ΔT^) mice collected at 8 dpi. Each dot represents an individual mouse. Error bars, SD. *P*-values, one-way ANOVA with a *post hoc* Tukey’s HSD test in panels **B and D–F**. The data were representative of two independent experiments (*n* = 4–9). (**C**) Representative fluorescence images of CD8^+^CD11b^+^ monocytes in the lungs of infected Ifngr1^ΔT^ mice shown in Fig. 4G. The slides were stained with anti-CD11b (green), anti-CD3 (magenta), and anti-CD8 (yellow) antibodies and DAPI (blue). Scale bar, 5 μm. (**D**) The percentages of CD8^+^Ly6C^+^ and CD8^-^Ly6C^+^ monocytes expressing GzmB in the lungs of naïve mice, and infected WT or *Ifngr1*^Δ^^T^ mice (*n* = 4–10). The percentages of CD8^+^Ly6C^+^ monocytes expressing IFN-γ (**E**) and the MFI in CD8^+^Ly6C^+^ monocytes (**F**) (*n* = 4–9). The IFN-γ staining was performed after PMA and ionomycin in the presence of GolgiStop. (**G**) The percentages of CD8^−^Ly6C^+^ monocytes (blue) or CD8^+^Ly6C^+^ monocytes (red) phagocyted RBCs infected with *P. berghei* ANKA parasites expressing mCherry (mCherry-PbANKA) *in vitro*. mCherry-PbANKA-iRBCs and lung cells isolated from mice infected with 10^4^ PbNK65-iRBCs at 8 dpi were co-cultured at a ratio of 5:1 for 90 min. (**H**) The percentages of CD8^−^Ly6C^+^ monocytes or CD8^+^Ly6C^+^ monocytes phagocyted mCherry-PbANKA-iRBCs *in vivo*. Mice infected with 10^4^ PbNK65-iRBCs were intravenously injected with 10^7^ mCherry-PbANKA-iRBCs at 8 dpi, and the lungs were isolated and assayed by flow cytometry 24 h after injection. Error bars, SD. *P*-values, Mann-Whitney U-test in (**G, H**). The data were representative of two independent experiments (*n* = 6). Total protein content (**I**) and cell count (**J**) in the BALF of infected mice at 8 dpi (*n* = 5–11). Each dot represents an individual mouse. Error bars, SD. *P*-values, one-way ANOVA with a *post hoc* Tukey’s HSD test. The mice were intravenously injected with 200 μL clodronate or control liposomes (5 mg/mL) 1 day before infection with 10^4^ PbNK65-iRBCs.

To further investigate the monocytic responses in *Ifngr1*^ΔT^ mice, we analyzed monocyte populations in the lungs of infected mice in detail and unexpectedly found the presence of a group of Ly6C^+^ monocytes expressing CD8 (Fig. 5A), and this group of monocytes was increased by 76% in the lungs of infected *Ifngr1*^ΔT^ mice compared to the infected WT mice (Fig. 5B). To confirm the presence of this population, we used two gating strategies (Fig. 5A; Fig. S8A through C) and confirmed that it was not due to the contamination by CD8^+^ DC, as no CD11c expression was observed in this population (Fig. S8D). Examining immunofluorescence images of the lung sections, we confirmed the presence of CD8^+^CD11b^+^ monocytes (Fig. 5C).

We found that the percentage of GzmB-expressing CD8^+^Ly6C^+^ monocytes was significantly higher than the CD8^−^Ly6C^+^ counterparts in the infected mice, increased by 24 times and 8 times in WT and *Ifngr1*^ΔT^ mice, respectively (Fig. 5D). And interestingly, both the percentage and expression level of IFN-γ in CD8^+^Ly6C^+^ monocytes were greatly reduced after infection in both *Ifngr1*^ΔT^ and WT mice (Fig. 5E and F).

We performed a co-culture of lung cells from PbNK65-infected WT mice at eight dpi with mCherry-expressing *P. berghei* ANKA (PbA) parasites or GFP-expressing *E. coli* at a ratio of 1:5 and 1:10, respectively, and examined the phagocytic capacity of CD8^+^Ly6C^+^ and CD8^-^Ly6C^+^ monocytes. Notably, higher percentages of CD8^+^Ly6C^+^ monocytes engulfed mCherry^+^ parasites or GFP^+^ bacteria compared to CD8^-^Ly6C^+^ monocytes (Fig. 5G; Fig. S9A through C). Additionally, we conducted an *in vivo* phagocytosis assay by intravenous injection of 10^7^ mCherry-expressing PbA parasites into PbNK65-infected WT mice at 8 dpi and analyzed the phagocytic capacity of CD8^+^Ly6C^+^ and CD8^−^Ly6C^+^ monocytes 24 h after parasite injection. The same as the *in vitro* assay, CD8^+^Ly6C^+^ monocytes uptook a higher (65%) percentage of mCherry^+^ parasites compared to CD8^−^Ly6C^+^ monocytes (Fig. 5H; Fig. S9C).

To further analyze the monocytic responses, we performed a macrophage/monocyte-depletion experiment using clodronate liposomes (30–32) 1 day before PbNK65 infection. Clodronate treatment in WT mice did not significantly affect the protein content in BALF (Fig. 5I), while the cell numbers in BALF were significantly reduced in the mice treated with clodronate compared to the mice treated with control liposomes (Fig. 5J). In sharp contrast, clodronate-treated *Ifngr1*^ΔT^ mice showed a 70% upregulation of protein content in BALF compared to the mice treated with control liposomes, reaching the level of control liposome-treated WT mice (Fig. 5H). Interestingly, no difference was observed for the cell numbers in BALF of *Ifngr1*^ΔT^ mice treated with clodronate or control (Fig. 5I).

Taken together, IFN-γ signaling in T cells contributes to regulating T cell-monocyte colocalization/interaction and monocyte responses. An increase of CD8^+^Ly6C^+^ proinflammatory monocytes was observed in the infected *Ifngr1*^ΔT^ mice, which exhibited higher phagocytic capacity and likely contributed to the lowered parasite load and ameliorated malaria-associated lung pathology in the infected *Ifngr1*^ΔT^ mice.

## DISCUSSION

**Fig 6:**
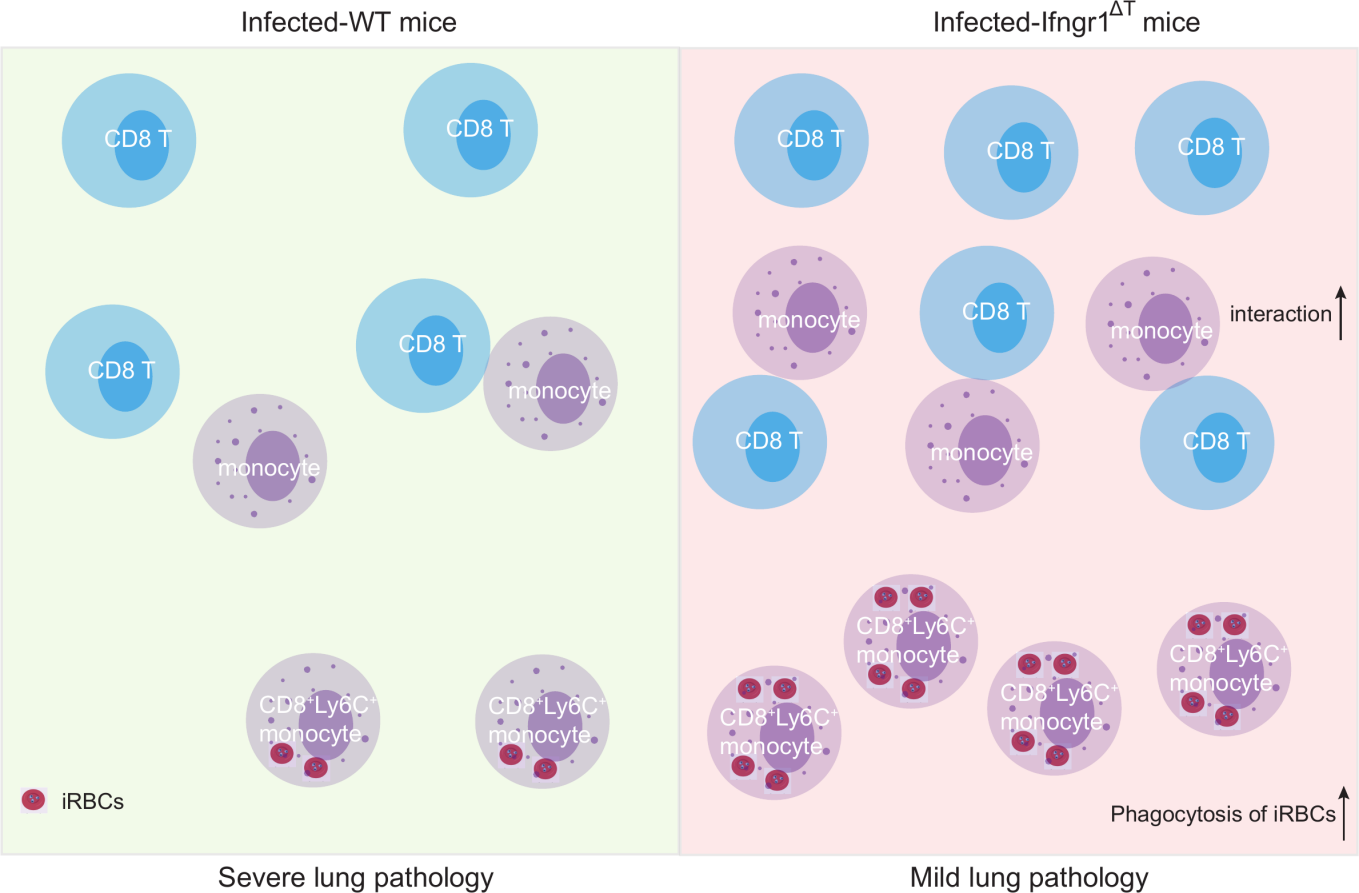
Disruption of IFN-γ signaling in T cells leads to attenuated lung pathology by enhancing T cell-monocyte interaction. With an infection dose of 10^4^ PbNK65-iRBCs, the *Ifngr1*^Δ^^T^ mice exhibited a milder lung pathology compared to the WT mice. In the infected *Ifngr1*^Δ^^T^ mice, the number of CD8^+^ T cells is increased, and the colocalization/interaction between CD8^+^ T cells and monocytes is enhanced. The fraction of proinflammatory (Ly6C^+^) monocytes expressing CD8 is increased, which shows heightened iRBC-phagocytic capability. iRBC, infected red blood cells.

In this study, we revealed that the deletion of IFN-γ signaling in T cells alleviates malaria-associated lung pathology by promoting T cell-monocyte interaction, specifically enhancing the phagocytic activity of proinflammatory monocytes in the *P. berghei* NK65-infected C57BL/6J mouse malaria model with a low (10^4^ iRBCs) infection dose (Fig. 6).

The role of IFN-γ in malaria control is debated. A meta-analysis reported that 37.5% of published studies showed that severe malaria patients had higher IFN-γ levels compared to uncomplicated malaria, while 58.3% of studies found no significant difference in IFN-γ levels between severe malaria and uncomplicated malaria patients (33). For the experimental models, the parasite line, infection dose, and mouse background all affect the results. In the non-lethal mouse malaria models, ablation of IFN-γ in mice resulted in much higher parasitemias and delayed parasite clearance of *P. yoelii* 17XL or *P. chabaudi* AS infection (34–36). In the lethal experimental cerebral malaria model, IFN-γ was reported to play a key role in brain endothelial cell activation and leukocyte recruitment in the brain during *P. berghei* ANKA infection (37–40). IFN-γ is also reported to contribute to malaria-associated lung pathology, as the IFN-γ knockout mice developed less lung pathology in both PbANKA (41) and PbNK65 infection models (25). Interestingly, IFN-γ depletion exhibited the same phenotypes as depletion of CD8^+^ T cells, indicating that either IFN-γ sourced from CD8^+^ T cells or the CD8^+^ T cell response to IFN-γ is critical in the mouse MA-ARDS model (42). Indeed, it was reported that CD3^+^ T cells accounted for 94% of IFN-γ production in the lungs of PbNK65-infected mice, with CD8^+^ T cells being the main source (25).

T cells play important roles during *Plasmodium* infection (43). CD8^+^ T cells mediated protection against liver-stage malaria (43); however, their roles in the blood stages are contentious. It was thought that CD8^+^ T cells contribute little to the control of the blood-stage infection, owing to the lack of MHC class I on erythrocytes (44). However, cytotoxic CD8^+^ T cells have the potential to target infected reticulocytes or immature erythroblasts that retain expression of MHC class I molecules (45). In a *P. chabaudi* mouse model, parasite-specific CD8^+^ T cells were required to control both acute and chronic blood stages (46). In contrast, CD8^+^ T cells were also reported to cause immunopathology in the brain and the lungs of mice upon recognition of cross-presented antigen on the vascular endothelial cells (37, 41). In this study, we also found that depletion of CD8^+^ T cells using anti-CD8β antibodies resulted in attenuated lung pathology.

CD4^+^CD25^high^Foxp3^+^ regulatory T cells (Tregs) were reported to suppress the functions of CD8^+^ T cells (47–49). Additionally, Tregs inhibit IFN-γ production in CD4^+^ T cells (50), thereby affecting IFN-γ signaling in other immune cells, including CD8^+^ T cells. Depletion of Tregs using anti-CD25 antibodies controlled parasitemias of *P. yoelii*-infected C57BL/6 mice only at the peak of the expansion phase (51), highlighting the complex immunoregulatory effect induced by *Plasmodium* parasites.

It was reported that IFN-γ knockout resulted in higher numbers of total and activated CD8^+^ T cells in a PbANKA mouse model (41). Similarly, we found that PbNK65-infected T cell-specific IFN-γR1 KO mice had higher numbers of CD8^+^ T cells and higher percentages of GzmB-expressing CD8^+^ T cells than WT controls. Importantly, the infected *Ifngr1*^ΔT^ mice developed lower parasitemias and attenuated lung pathology compared to the WT mice. Using spatial transcriptomics and immunofluorescence analyses, we found that the pathways such as “leukocyte cell-cell adhesion” and “leukocyte migration” were activated and the colocalization of T cell-monocyte was enhanced in the lungs of infected *Ifngr1*^ΔT^ mice.

Not only do monocytes activate T cell functions (52), but T cells or T cell-monocyte interactions also impact monocyte responses. For example, in an atherosclerosis model, depletion of CD8^+^ T cells decreases circulating Ly6C^high^ monocytes (53). Interestingly, it was shown that T cells contribute to the trained immunity in human monocytes in response to *P. falciparum*-iRBCs (54, 55). In our study, the enhanced T cell-monocyte colocalization/interaction in the infected *Ifngr1*^ΔT^ mice was associated with a heightened phagocytic capacity of monocytes and reduced the parasite loads in the lungs. Whether the elevated monocyte response is due to T cell-mediated trained immunity in monocytes requires further investigation.

In addition, we identified an increase in a subgroup of proinflammatory monocytes in the lungs of the infected *Ifngr1*^ΔT^ mice, characterized by the expression of both Ly6C and CD8. CD8^+^ monocytes/macrophages were previously reported in humans and rats, which had enhanced expression of cytotoxic mediators, such as Fas ligand, perforin, and granzymes, and increased abilities of cytotoxicity and tumor cell killing (56–60). We also found that CD8^+^Ly6C^+^ monocytes had higher GzmB expression, in line with previous reports. Due to the limited numbers, we were unable to perform adoptive transfer for this subset of monocytes; however, the *in vitro* and *in vivo* phagocytic assays showed that CD8^+^Ly6C^+^ monocytes had higher phagocytic activity. In addition, depletion of macrophages/monocytes using clodronate in the infected *Ifngr1*^ΔT^ mice resulted in the upregulated protein content in the BALF reaching the level of WT control mice without clodronate depletion, suggesting that the elevated monocytic response contributed to the attenuated lung pathology in the *Ifngr1*^ΔT^ mice.

## MATERIALS AND METHODS

### Mice

C57BL/6J mice aged between 7 and 12 weeks were purchased from Beijing HFX Biotechnology Company (Beijing, China) and accommodated for at least 7 days in the animal facilities before experiments. *Ifngr1*^fl/fl^ and *GzmB*^fl/fl^ were purchased from Cyagene (Jiangsu, China), and Lck-iCre transgenic mice were purchased from Nanjing Biomedical Research Institute of Nanjing University (Jiangsu, China). T cell-specific *Ifngr1* knockout (*Ifngr1*^fl/fl^ Lck-iCre^+^, *Ifngr1*^ΔT^) or *GzmB* knock-out (*GzmB*^fl/fl^ Lck-iCre^+^, *GzmB*^ΔT^) mice and control wild-type mice (*Ifngr1*^+/+^ Lck-iCre^+^, *Ifngr1*^WT^; *GzmB*^+/+^ Lck-iCre^+^, *GzmB*^WT^) were generated by crossing *Ifngr1*^fl/fl^ mice or *GzmB*^fl/fl^ mice with Lck-iCre transgenic mice. All transgenic mice used in the comparison studies were matched for age and sex. Primers for genotyping are listed in Table S2. Mice were housed and bred under SPF conditions (Specific Pathogen Free) at the Laboratory Animal Center of West China Second University Hospital.

### Parasites and infection

*P. berghei* NK65 (PbNK65) and mCherry-expressing *P. berghei* ANKA (PbA 1868cl1, mCherry is under the control of HSP70 promoter) were kindly provided by Dr. Chris J. Janse, Leiden University. The infections were performed by i.p. injection of 10^4^ or 10^6^ PbNK65-iRBCs. The mouse condition was closely monitored after infection. Parasitemia was monitored on Giemsa-stained thin blood films by enumerating the percentage of RBCs infected with asexual parasites. The animals were euthanized upon reaching humane endpoints, exhibiting signs of hyperparasitemia (over 85%) or severe hypothermia (body temperature below 28°C). The infection experiments were conducted at the designated region in the Laboratory Animal Center of West China Second University Hospital.

### Lung pathology analysis

Mice were terminally anesthetized with 0.2 mL 3% pentobarbital (Shanghai Rongbai Biological Technology, #P8410-5), and the lungs were cannulated and inflated with 0.5 mL cold PBS (Solarbio, #P1020). The BALF was collected by centrifuging the total fluid at 500 g at 4°C for 10 min. Red blood cells were lysed with Red Blood Cell Lysis Buffer (Solarbio, #R1010), and the remaining cells were enumerated. The protein content in BALF was measured using Pierce BCA Protein Assay Kit (Thermo, #23227) according to the manufacturer’s instructions and quantified on an Infinite M200 Pro (Tecan).

Evans blue powder (Sangon Biotech, #A602025) was dissolved to 50 mg/mL in PBS and filtered through a 0.22 μm filter. The solution was intravenously (i.v.) injected into mice (50 mg/kg). Mice were terminally anesthetized with an injection of 0.2 mL 3% pentobarbital 120 min after Evans blue injection. The lungs were extensively perfused using 20 mL PBS. The collected lungs were weighed, dehydrated for 48 h at 65°C in the dark, and placed in formamide (8 mL/g) (Sigma, #SLBZ073) for another 48 h in the dark. The extracted Evans blue was quantified at an absorbance of 620 nm with Infinite M200 Pro (Tecan). The standard curve method was used to calculate the content of Evans blue (61).

For histological examination, the lungs were collected after extensive transcardiac perfusion with 10 mL PBS. The lungs were inflated and fixed with 4% PFA (Biosharp, #C0040) for 24 h, dehydrated using 75% ethanol (Chron Chemicals, #64-17-5), embedded in paraffin, and sectioned. The sections were stained with H&E (Solarbio, #G1120) and photographed under a Pannoramic MIDI scanner system (3DHISTECH, Budapest, Hungary) with a 20× objective lens.

### Multiplexed immunofluorescence

Formalin-fixed paraffin-embedded lung sections (4 μm) prepared on a Leica RM2235 were baked for 1 h at 62°C followed by deparaffinization with Xylene (Sinopharm Chemical Reagent Co., Ltd., #10023418), rehydration with ethanol (Sinopharm Chemical Reagent Co., Ltd., #100092683), and antigen retrieval in Tris-EDTA solution (Tris-base Sigma, #648310; EDTA, Sigma, #E9884; PH = 9.0) for 24 min at boiled state, and incubated in 3% H_2_O_2_ solution (Sinopharm Chemical Reagent Co., Ltd., #73113760) for 10 min at room temperature to reduce nonspecific background. Immunofluorescence staining using tyramide signal amplification kit (Runnerbio, 488 Tyramide, Bry-880488; CY3 Tyramide, Bry-880CY3, and CY5 Tyramide, Bry-880CY5) was performed in three cycles with antibodies of anti-mouse CD11b (Abcam, #ab133357, 1:2,000), CD3 (Abcam, #ab16669, 1:200), and CD8 (Abcam, #ab217344, 1:1,000). In each cycle, the lung sections were incubated with a 100 μL primary antibody for 12 h at 4°C and 1 h at 37°C, followed by a 100 μL secondary antibody for 1 h at 37°C and 100 μL CY3 Tyramide/Cy5 Tyramide/488 Tyramide for 30 min at room temperature. After nuclear counterstaining with DAPI, the slides were coverslipped with VECTASHIELD Vibrance (VECTOR, #ZE1011). The slides were scanned using PANNORAMIC SCAN II Digital Scanner (3DHISTECH) with a 20× objective lens. The images were analyzed at 20× magnification using the CaseViewer 2.4 software (62).

T cell-monocyte colocalization was quantified as the average rate of (CD8^+^) CD3^+^ T cells colocalized with CD11b^+^ monocytes or monocytes colocalized with T cells in 20 randomly picked fields-of-view (FOVs; 282 × 176 μm^2^) in the lung sections. FOV selection avoided big air tubes and blood vessels.

### Flow cytometry

Lungs were collected after transcardiac perfusion, diced, and digested using 2 μg/mL Liberase (Thermo, #32955) in RPMI containing 5% fetal bovine serum (FBS; Cell-Box, #SV30087.03) and 25U/mL DNase I (Roche, #11284932001) at 37°C for 30 min. The digested lung tissues were passed through 70 μm cell strainers (Falcon, #352350) and washed using PBS containing 1% FBS. RBCs were lysed using Red Blood Cell Lysis Buffer at 4°C for 5 min, and the remaining cells were washed twice with 200 μL PBS and counted using a hemocytometer. To confirm the knockout efficiency of *Ifngr1*^ΔT^, the spleens were collected and diced into small pieces, passed through 70 μm cell strainers, and pelleted at 500 × *g* for 5 min at 4°C. RBCs were lysed using Red Blood Cell Lysis Buffer at 4°C for 5 min, and the remaining cells were washed twice with 200 μL PBS and counted using a hemocytometer. To confirm the removal efficiency of CD8^+^ T cells after antibody depletion described below, the peripheral blood was collected and lysed with Red Blood Cell Lysis Buffer at 4°C for 5 min and the remaining cells were washed twice with 200 μL PBS before antibody staining.

The prepared single-cell suspension was incubated with Mouse Fc Block (BD, #553142) for 20 min, followed by live/dead staining using Fixable Viability Stain 570 (BD, #564995) or Fixable Viability Stain 520 (BD, #564407) and surface staining with fluorochrome-labeled antibodies as follows: BV510 anti-mouse CD19 (BD, #562956; clone 1D3), BV605 anti-mouse CD4 (BD, #563151; clone RM4-5), PE anti-mouse CD3e (BD, #553064; clone 145-2C11), PE anti-mouse TER-119/erythroid cells (BD, #553673; clone TER119), BV510 anti-mouse CD11c (Biolegend, #117338; clone N418), BV711 anti-mouse CD11b (BD, #563168; clone M1/70), BV711 anti-mouse CD19 (BD, #563157; clone 1D3), AlexaFluor 700 anti-mouse CD4 (BD, #557956; clone RM4-5), BV711 anti-mouse CD119 (BD, #740706; clone GR20), BV605 anti-CD4 (BD, #563151; clone RM4-5), FITC anti-mouse TER-119/erythroid cells (Biolegend, #116206; clone TER119), PE anti-mouse CD19 (Biolegend, #115508; clone 6D5), BV421 anti-mouse F4/80 (Biolegend, #123137; clone BM8), PerCp/cyanine 5.5 anti-mouse Ly6G (Biolegend, #127616; clone IA8), PE/cyanine 5 anti-mouse CD8a (Biolegend, #100710; clone 53-6.7), PerCp/cyanine 5.5 anti-mouse CD3e (Biolegend, #100328; clone 145-2C11), PE/cyanine 7 anti-mouse CD31 (Biolegend, #102418; clone 390), FITC anti-mouse CD45 (Biolegend, #103108; clone 30-F11), PE/cyanine 5 anti-mouse I-A/I-E (Biolegend, #107612; clone M5/114.15.2), PerCP/cyanine 5.5 anti-mouse I-A/I-E (Biolegend, #107626; clone M5/114.15.2), FITC anti-mouse CD170 (Siglec-F) (Biolegend, #155504; clone S17007L), PE/cyanine 7 anti-mouse CD3e (Biolegend, #100320; clone 145-2C11), APC/cyanine 7 anti-mouse CD3e (Biolegend, #100330; clone 145-2C11), BV605 anti-mouse Ly-6C (Biolegend, #128036; clone HK1.4), BV421 anti-mouse Ly-6C (Biolegend, #128032; clone HK1.4), BV510 anti-mouse CD3 (Biolegend, #100234; clone 17A2), PE/Dazzle 594 anti-mouse CD8a (Biolegend, #100761; clone 53-6.7), PE/Dazzle 594 anti-mouse CD45 (Biolegend, #103146; clone 30-F11), PE/cyanine 5 anti-mouse CD45 (Biolegend, #103109; clone 30-F11), PE/cyanine 7 anti-mouse Ly-6G (Biolegend, #127617; clone I8A), APC/cyanine 7 anti-mouse Ly6G (Biolegend, #127623; clone I8A), APC anti-mouse CD11c (Biolegend, #117310; clone N418), FITC anti-mouse CD170 (SiglecF, Biolegend, #155525; clone S17007L). Details of flow panels are listed in Table S1.

For IFN-γ staining, the cells were first resuspended in RPMI 1640 containing 5% FBS and 1% penicillin-streptomycin (Gibco, #15140-122) to a concentration of 1–2 × 10^6^ cells/mL, and treated with 1 μmol/mL PMA (Yeasen, #5061ES03) and 1 μg/mL ionomycin (Yeasen, #56092-81-1) for 4–6 h in the presence of GolgiStop protein transport inhibitor (BD, #554724) at 37°C with 5% CO_2_. After stimulation, the cells were collected for surface and subsequent intracellular staining. The surface-stained cells were fixed and permeabilized using the Fixation/Permeabilization intracellular staining kit (BD Bioscience, #554714). After washing twice with PBS, the cells were then incubated with intracellular staining antibodies, including PE/cyanine 7 or FITC anti-human/mouse granzyme B (GzmB; Biolegend, #372214 or 372206; clone QA16A02), BV786 anti-mouse Ki-67 (BD, #563756; clone B56), or BV786 anti-mouse IFN-γ (BD, #563773; clone XMG1.2). The stained cells were analyzed on an Attune Nxt flow cytometer (Thermo, #AFC2-4486520) or Cytek Aurora CS (Cytek). Data were analyzed using FlowJo V10 (BD).

Details of flow panels are listed in Table S1. The gating strategies for lung adaptive cells are shown in Fig. S1E (lung panel 1) and Fig. S1H (lung panel 2). The gating strategy of lung innate cells is shown in Fig. S2A (lung panel 3). The gating strategy of lung immune cells with intracellular staining is shown in Fig. S7A. Fluorescence minus one controls of lung panel 5 were shown in Fig. S7B.

### *In vitro* phagocytosis assay

The lungs of wild-type C57BL/6J i.p. injected with 10^4^ iRBCs of PbNK65 were isolated at 8 dpi as previously described and resuspended to 2 × 10^6^ cells/mL with RPM1640 containing 10% FBS and 1% penicillin-streptomycin. The cells were then co-cultured with GFP-expressing *E. coli* at a ratio of 10:1 or with mCherry-expressing *P. berghei* ANKA at a ratio of 5:1 for 90 min at 37°C with 5% CO_2_. The cells were collected and incubated with Mouse Fc Block (BD, #553142) for 20 min at 4°C, followed by staining with live/dead cell stains and surface staining fluorochrome-labeled antibodies for flow cytometry analysis as described before. The gating strategies for the phagocytosis assay are shown in Fig. S9C.

### *In vivo* phagocytosis assay

C57BL/6J mice were first i.p. infected with 10^4^ PbNK65-iRBCs. At 8 dpi, the infected mice were i.v. injected with 10^7^ mCherry-expressing PbA parasites. After 24 h, the lungs of infected mice were isolated for flow cytometry analysis as described before. Gating strategies for the assay were shown in Fig. S9C.

### Treatment of mice with anti-CD8β

The mice were i.v. injected with 50 μg anti-CD8β antibody (BioXCell, clone 53-5.8, #BE0088) or antibody isotype control (BioXCell, clone HRPN, #BE0223) 1 day before infection with 10^4^ PbNK65-iRBCs. At 4 dpi, the infected mice were treated with 20 μg anti-mouse CD8β antibody or antibody isotype control, and the BALF was collected at 8 dpi. For the mice used to follow parasitemias, an extra injection was performed at 8 dpi and the depletion efficiency was maintained until 17 dpi (Fig. S5A). The antibodies were freshly diluted in PBS (pH 7.0) without stabilizers or preservatives before use.

### Treatment of mice with clodronate

The mice were i.v. injected with 200 μL clodronate liposomes (YEASEN, #40337ES08) or control liposomes (YEASEN, #40338ES08) to analyze the effect of monocyte/macrophage depletion as published before (31). A day after liposome injection, the mice were infected with 10^4^ PbNK65-iRBCs, and the BALF was collected at 8 dpi.

### RNA extraction and RT-qPCR

After extensive transcardiac perfusion, two lobes of the lungs were collected into 1 mL TRI Reagent solution (Invitrogen, #9738G) and homogenized with a tissue lyser (SCIENTZ, #SCIENTZ-48). Total RNAs were extracted using the GeneJET RNA Purification Kit (Thermo, #EB27FA001) according to the manufacturer’s recommendations. The concentration and purity were evaluated using a NanoDrop spectrophotometer (Nanodrop 2000, Thermo Fisher Scientific). RT-qPCR was performed using SYBR Green Master Mix (Yeasen, #11184ES08) on a Bio-Rad CFX96 real-time PCR apparatus (BIO-RAD, #7B8BR07672). Primer sequences were listed in Table S2.

For RNA to be used for sequencing, the RNA integrity and concentration were analyzed using an Agilent 2100 Bioanalyzer (Agilent) and a Qubit 2.0 (Invitrogen, #Q33226), respectively. cDNA was obtained using the RevertAid RT Reverse Transcription Kit (Thermo Fisher Scientific, #K1622).

### Time-series transcriptomics analysis of lung tissues

RNAs of the lungs were extracted using MiRNeasy Micro Kit (QIAGEN, #1071023) according to the manufacturer’s recommendation. All samples with an RNA integrity number above seven were used for library preparation and subsequent RNA-seq. The sequencing library was prepared using Illumina TruSeq PE Cluster Kit V3-cBot-HS and sequenced on an Illumina HiSeq platform (Illumina, San Diego, CA, USA), yielding 125 bp/150 bp paired-end chain-specific reads.

The Fastq files first underwent a quality assessment using FastQC (v.0.12.1, https://www.bioinformatics.babraham.ac.uk/projects/fastqc/). The raw reads were then trimmed with Trimmomatic (v.0.39) (63) to remove low-quality reads and adapter sequences. The cleaned reads were aligned to the mouse reference genome (mm10) utilizing STAR (v.2.5.3) (64) with default parameters. Subsequently, the quantification of the aligned reads was performed using the GenomicAlignments (v.1.24.0) (65) package. Genes with total raw expression levels below 10 were removed across all samples. Subsequently, the quality-controlled gene expression matrix was normalized using the DESeq2 (v.1.24.0) (66) package.

For gene clustering analysis, Mfuzz (v.2.48.0) (26) was used to perform gene expression clustering analysis on the RNA-seq data. First, we extracted the normalized gene expression matrix from the DESeqDataSet object. Next, we constructed an ExpressionSet object using the “new” function and standardized it with the “standardise” function. Finally, we applied the “mfuzz” function to cluster the genes.

The composition of immune cells was analyzed using xCell (v.2.48.0) (67). We applied the xCellAnalysis function after extracting the normalized gene expression matrix from the DESeqDataSet object.

Gene set enrichment scores for each sample were calculated using the “gsva” function of the GSVA R package (v.1.36.3) (68). First, we extracted the normalized gene expression matrix from the DESeqDataSet. The gsva function was then applied with the following parameters: kcdf = “Gaussian,” method = “ssgsea,” mx.diff = 1, and parallel.sz = 1.

DEG analysis was performed using the DESeq2 package (v.1.24.0) (66), which employs a negative binomial distribution model to analyze gene expression data, estimates gene dispersion, and uses a Wald test or likelihood ratio test to identify significant changes in gene expression. DEGs were identified by applying the “results” function with the criteria of |log2(fold change)| > 2 and adjusted *P*-value <0.05. *P*-values were adjusted using the BH method.

### Analysis of single-cell RNA-seq data

The scRNA-seq data of naïve and PbNK65-infected lungs (23) were downloaded from the NCBI Gene Expression Omnibus database under the accession number GSE244528 (23). First, the count matrices were imported using the Read10× function available in Seurat (v.4.1.1) (69). Secondly, the raw counts were subjected to normalization using the “LogNormalize” function. Next, highly variable genes were identified using the “FindVariableFeatures” function, followed by scaling the normalized data to Z-scores with the “ScaleData” function. Subsequently, principal components were calculated from the scaled data using RunPCA. Next, the “FindNeighbors” function was applied to construct a k-nearest neighbor graph, which refined the edge weights between cell pairs. Uniform manifold approximation and projection (UMAP) was performed for dimensionality reduction with the “RunUMAP” function. Clustering of the cells was carried out using the “FindClusters” function, with the original Louvain algorithm. Markers for each identified cluster were determined using the “FindAllMarkers” function in Seurat, with parameters set as min.pct = 0.1, logfc.threshold = 0.25, and only.pos = TRUE. Classical markers for immune cells, epithelial cells, and fibroblasts were used to annotate cell clusters.

For cell-cell communication analysis, the R package NicheNet (v.2.1.5) (28) was used to analyze cell communication, using T cells as the “receiver” and the rest of the cells as the “senders.” scRNA-seq data of naïve and PbNK65-infected lungs were analyzed to investigate differences in T cell communication before and after infection. Samples were divided into two groups: the uninfected group, defined as “Naïve,” and the infected group, defined as “Infected.” By employing the “nichenet_seuratobj_aggregate” function with parameters set as sender = “all,” receiver = “T cells,” condition_colname = “Group,” condition_oi = “Infected,” condition_reference = “Naïve,” and expression_pct = 0.05.

Seurat (v.4.1.1) (69) package was used to perform DEG analysis for scRNA-seq data. The data were first normalized using the “NormalizeData” function with the LogNormalize algorithm. The “FindMarkers” function was then used for differential expression analysis with the criteria of |log2(fold change)| > 0.25 and adjusted *P*-value < 0.05. *P*-values were derived using the Wilcoxon test with Bonferroni correction.

### Spatial transcriptomics

Spatial transcriptomics Gene Expression Kit (BMKMANU, #ST03002) and Tissue Optimization Kit (BMKMANU, #ST03003) were used according to manufacturer instructions. Each capture area of the gene expression slide (6.8 × 6.8 mm^2^) contains 2,200,000 barcoded spots that are 2.5 μm in diameter (4.8 μm center to center between spots), providing an average of 3 to 6 cells per spot. The frozen tissue was then embedded in an Optimal Cutting Temperature compound (OCT, Sakura Tissue-TEK) on dry ice and stored at −80°C. Before performing the complete protocol, the Tissue Optimization Kit was used according to the manufacturer’s instructions, and the fluorescent footprint was imaged using a Metafer Slide Scanning Platform (Pannoramic MIDI) to select the optimal permeabilization time. OCT blocks were cut with a pre-cooled cryostat at 10 μm thickness, and sections were transferred to fit the 6.8 × 6.8 mm^2^ oligo-barcoded capture areas on the BMKMANU S1000 Gene Expression Slide. The Gene Expression Slide with tissues was fixed and stained with H&E and imaged using a Pannoramic MIDI microscope at 40× magnification. The Pannoramic MIDI was used to acquire tile scans of the entire array and merge images. Sequence libraries were then processed according to the manufacturer’s instructions (BMKMANU, Library Construction Kit, #ST03002-34). Libraries were prepared with TruSeq Illumina libraries and sequenced on a NovaSeq (Illumina) at a minimum sequencing depth of 150,000 read pairs per spatial spot using Integragen (Evry). Sequencing was performed with the recommended protocol (read 1: 28 cycles; i7 index read: 10 cycles; i5 index read: 10 cycles; and read 2: 50 cycles), yielding 514.85 million sequencing reads.

FASTQ files and manually aligned histology images were analyzed using BSTMatrix (v.2.3j, http://www.bmkmanu.com/portfolio/tools). Then the data were mapped to the mouse reference genome (mm10) using the STAR genome aligner version v.2.5.1b. Processed data were imported into R via Seurat (v.4.1.1) (69) for detailed data filtering, normalization, and visualization. For quality control, high-quality spots were retained based on specific criteria, specifically those with 1,000 < UMI count < 5,000 and 600 < gene count < 2,500. Consequently, a total of 7,636 spots from naïve mouse, 11,219 spots from WT-infected, and 20,054 spots from *Ifngr1*-KO-infected mouse were included for downstream analysis. Finally, data normalization was performed on independent tissue sections using the variance-stabilizing transformation method implemented in the “SCTransform” function of Seurat.

To examine the spatial distribution of immune cells, RCTD (v.2.2.1) (70) was employed to deconvolute the transcriptomic data of each spot into potential cell types. Initially, the “Reference” function was applied to build a reference object, while the “SpatialRNA” function was used to create a spatial RNA object for deconvolution. Next, the “create.RCTD” function was utilized to generate an RCTD object by combining the reference object and the spatial RNA object. The run.RCTD function was used for deconvolution, with the doublet_mode parameter set to “full.” We next qualitatively assessed the presence of immune cells potentially in each spot. If the content value of a particular immune cell type in a spot exceeded the average content of that cell type across all samples, we considered that spot to potentially harbor the immune cell type. Spots containing both immune cell types were defined as “colocalization”.

For DEG analysis, the data were normalized using the “SCTransform” function. Prior to differential expression analysis, the “PrepSCTFindMarkers” function was applied to the normalized matrix, followed by differential expression analysis using the “FindMarkers” function with the criteria of log2(fold change) > 0 and adjusted *P*-value < 0.05. *P*-values were derived using the Wilcoxon test with Bonferroni correction.

Enrichment analyses were performed on differentially expressed genes using the “enrichGO” and “enrichKEGG” functions from the ClusterProfiler R package (v.4.9.0.002) (71). For GSEA, the “gseGO” function from the org.Mm.eg.db (v.3.18.0) package was used, and the genes were ranked based on their log2(fold change). Visualization was conducted using the GseaVis package (v.0.0.5, https://github.com/junjunlab/GseaVis). Pathways with adjusted *P*-values <0.05, corrected by the BH method, were selected for visualization.

### Statistical analysis

All bioinformatics analyses were performed using R (v.4.2.1). For animal experiments, graphs were generated using the Prism program (GraphPad software 10, San Diego, CA, USA).

## Data Availability

The codes and the processed time-series RNA-seq, scRNA-seq, and spatial transcriptomics data used in this study are available on GitHub (https://github.com/LuChenLab/PbNK65). The raw bulk RNA-seq data for naïve and infected samples were deposited in the NCBI BioProject repository under accession number GSE279789, and the raw spatial transcriptomics data are available under accession number GSE283333.

## SUPPLEMENTAL MATERIAL

Supplemental figures mbio.03938-25-s0001.pdf Figures S1 to S9.

Table S1 mbio.03938-25-s0002.xlsx Antibodies and panel details used for flow cytometry.

Table S2 mbio.03938-25-s0003.xlsx Primers used in this study.

Table S3 mbio.03938-25-s0004.xlsx Differentially expressed genes in the lungs of PbNK65-infected mice compared to naïve mice.

Table S4 mbio.03938-25-s0005.xlsx The enriched pathways in the lungs of PbNK65-infected mice compared to naïve mice.

Table S5 mbio.03938-25-s0006.xlsx Gene clusters and related pathways based on expression profiles during infection.

Table S6 mbio.03938-25-s0007.xlsx Differentially expressed genes in different cell types in the lungs of mice developed MA-ARDS.

Table S7 mbio.03938-25-s0008.xlsx Highly expressed genes and related pathways in different regions identified in spatial transcriptomics.

ASM does not own the copyrights to Supplemental Material that may be linked to, or accessed through, an article. The authors have granted ASM a non-exclusive, world-wide license to publish the Supplemental Material files. Please contact the corresponding author directly for reuse.
