# Supplementary material for: Comprehensive immune profiling reveals IFN-γ signaling in T cells mediates parasite phagocytosis in a rodent malaria model
Source: mBio. 2026 Mar 11;17(4):e03938-25. doi: 10.1128/mbio.03938-25 (PMC13059739; doi:10.1128/mbio.03938-25)
Supplement: Supplemental figures — Figures S1 to S9. [file mbio.03938-25-s0001.pdf]

FIG S1

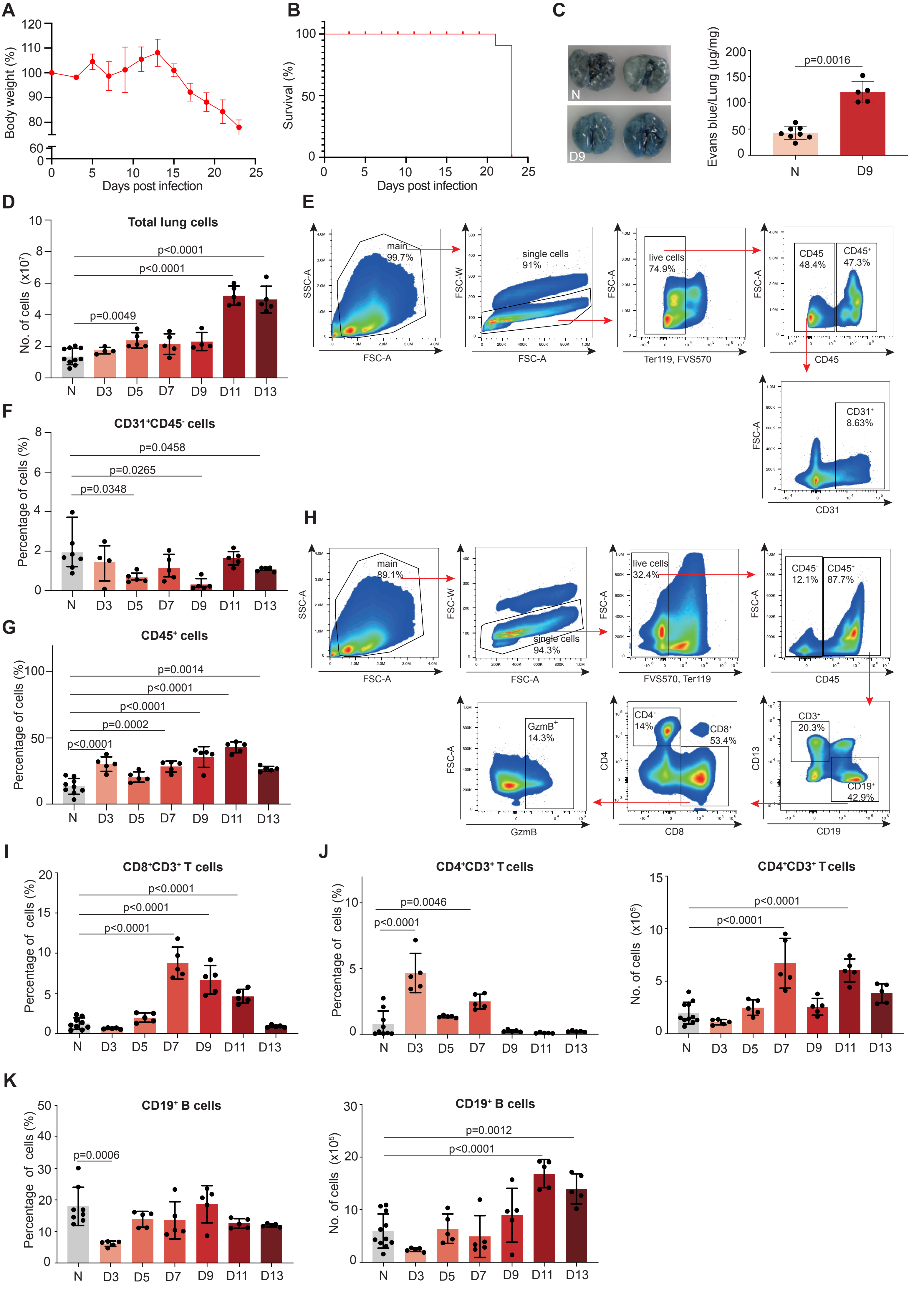

A
Body weight (%)
Days post infection
B
Survival (%)
Days post infection
C
N
D9
Evans blue/Lung (μg/mg)
p=0.0016
D
Total lung cells
No. of cells (x10⁷)
p<0.0001
p<0.0001
p=0.0049
N D3 D5 D7 D9 D11 D13
E
main 99.7%
single cells 91%
live cells 74.9%
CD45⁻ 48.4%
CD45⁺ 47.3%
CD31⁺ 8.63%
SSC-A
FSC-A
FSC-W
Ter119, FVS570
CD45
CD31
F
CD31⁺CD45⁻ cells
Percentage of cells (%)
p=0.0458
p=0.0265
p=0.0348
G
CD45⁺ cells
Percentage of cells (%)
p=0.0014
p<0.0001
p<0.0001
p=0.0002
p<0.0001
H
main 89.1%
single cells 94.3%
live cells 32.4%
CD45⁻ 12.1%
CD45⁺ 87.7%
CD3⁺ 20.3%
CD19⁺ 42.9%
CD4⁺ 14%
CD8⁺ 53.4%
GzmB⁺ 14.3%
FVS570, Ter119
CD13
CD19
CD4
CD8
GzmB
I
CD8⁺CD3⁺ T cells
Percentage of cells (%)
p<0.0001
p<0.0001
p<0.0001
J
CD4⁺CD3⁺ T cells
Percentage of cells (%)
p=0.0046
p<0.0001
CD4⁺CD3⁺ T cells
No. of cells (x10⁵)
p<0.0001
p<0.0001
K
CD19⁺ B cells
Percentage of cells (%)
p=0.0006
CD19⁺ B cells
No. of cells (x10⁵)
p=0.0012
p<0.0001

FIG S1. Pathology and flow cytometry analyses of the infected mice.

(**A**) The change of body weight and (**B**) survival rate of PbNK65-infected C57BL/6J mice (n=6). Means with SD were shown in the line chart. (**C**) The representative images and the amount of Evans blue in the lungs of naïve (N) or infected mice collected at 9-day post infection (D). Each dot represents a biological replicate (n=5–8). Error bar, SD. P-values, Mann-Whitney U test. (**D**) The total cell counts of the lungs of naïve or infected mice at different days post infection (n=4–10). Error bar, SD. P-values, One-way ANOVA with a Post Hoc Tukey's HSD test in (D, F-G, I-K). Naïve samples were collected at different time points together with the infected samples to ensure the accuracy of the analysis. (**E**) Gating strategy of the endothelial cells and immune cells. Percentages of $CD31^+CD45^-$ cells (**F**) and $CD45^+$ cells (**G**) in the lungs of naïve and infected mice (n=5–9). (**H**) Gating strategy of the adaptive immune cells in the lungs. (**I**) The percentages of $CD8^+CD3^+$ T cells in the lungs of naïve or infected mice (n=5–9). The percentages and cell numbers of $CD4^+CD3^+$ T cells (**J**) and $CD19^+$ B cells (**K**) in the lungs (n=5–11).

FIG S2

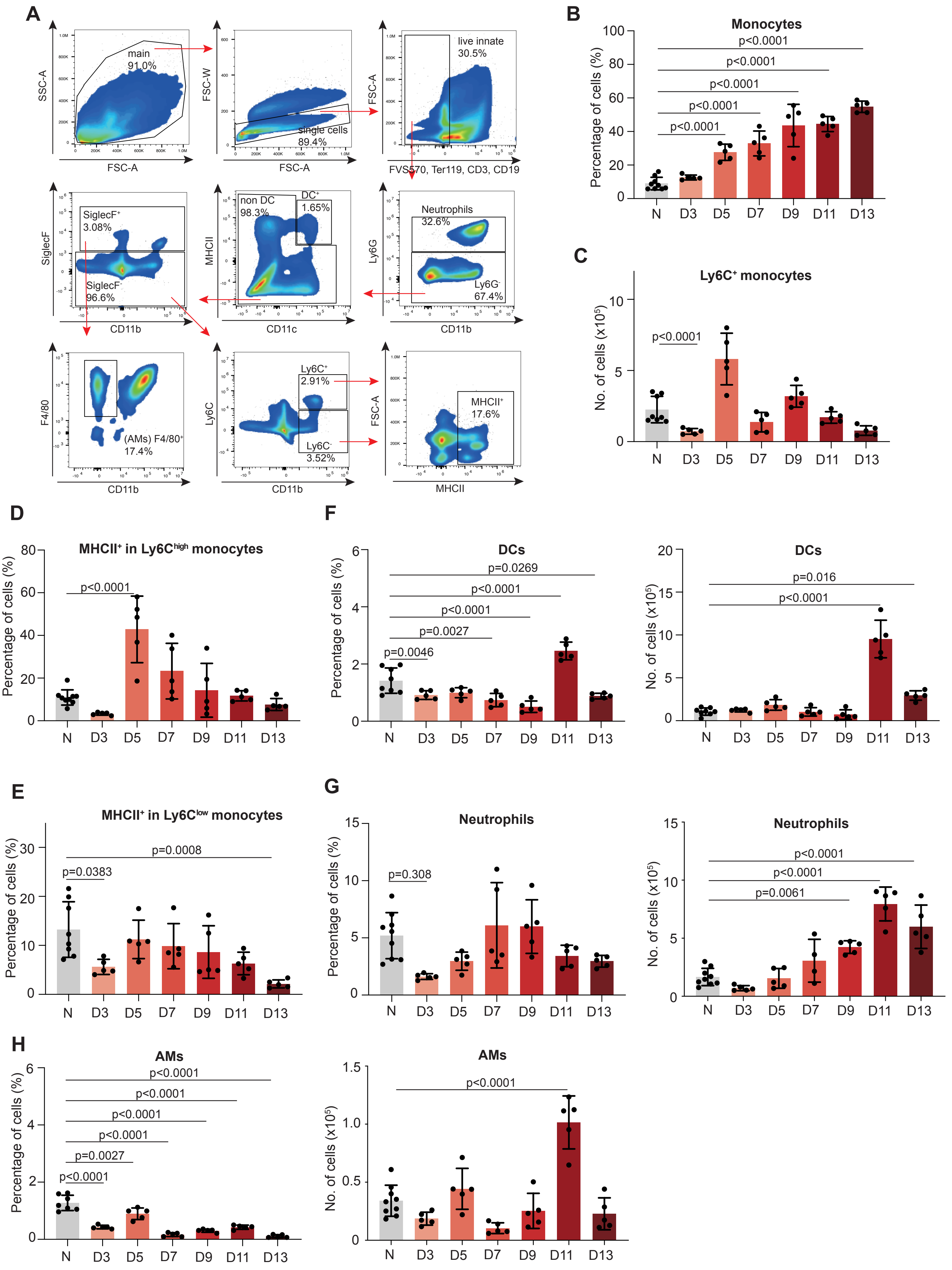
A
SSC-A
FSC-A
main
91.0%
FSC-W
single cells
89.4%
live innate
30.5%
FVS570, Ter119, CD3, CD19
SiglecF
CD11b
SiglecF+
3.08%
SiglecF-
96.6%
MHCII
CD11c
non DC
98.3%
DC+
1.65%
Ly6G
Neutrophils
32.6%
Ly6G-
67.4%
F4/80
(AMs) F4/80+
17.4%
Ly6C
Ly6C+
2.91%
Ly6C-
3.52%
MHCII+
17.6%
B
Monocytes
Percentage of cells (%)
p<0.0001
p<0.0001
p<0.0001
p<0.0001
p<0.0001
N
D3
D5
D7
D9
D11
D13
C
Ly6C+ monocytes
No. of cells (x10^5)
p<0.0001
D
MHCII+ in Ly6Chigh monocytes
Percentage of cells (%)
p<0.0001
F
DCs
Percentage of cells (%)
p=0.0269
p<0.0001
p<0.0001
p=0.0027
p=0.0046
DCs
No. of cells (x10^5)
p=0.016
p<0.0001
E
MHCII+ in Ly6Clow monocytes
Percentage of cells (%)
p=0.0008
p=0.0383
G
Neutrophils
Percentage of cells (%)
p=0.308
Neutrophils
No. of cells (x10^5)
p<0.0001
p<0.0001
p=0.0061
H
AMs
Percentage of cells (%)
p<0.0001
p<0.0001
p<0.0001
p<0.0001
p=0.0027
p<0.0001
AMs
No. of cells (x10^5)
p<0.0001

FIG S2. Flow cytometry analysis of the innate immune cells in the lungs. (**A**) Gating strategy of the innate immune cells in the lungs. The percentage of monocytes (**B**) and the number of $Ly6C^+$ monocytes (**C**) in the lungs of naïve (N) and infected mice (n=5–9). Naïve samples were collected at different days post infection (D) together with the infected samples to ensure the accuracy of the analysis. Each dot represents an individual mouse. Error bar, SD. P-values, One-way ANOVA with a Post Hoc Tukey's HSD test in (B-H). The percentages of $MHCII^+$ cells in $Ly6C^{high}$ (**D**) and $Ly6C^{low}$ (**E**) monocytes (n=5–9). The percentages and cell numbers of dendritic cells (DCs) (**F**), neutrophils (**G**) and alveolar macrophages (AMs) (**H**) (n=5–9).

FIG S3

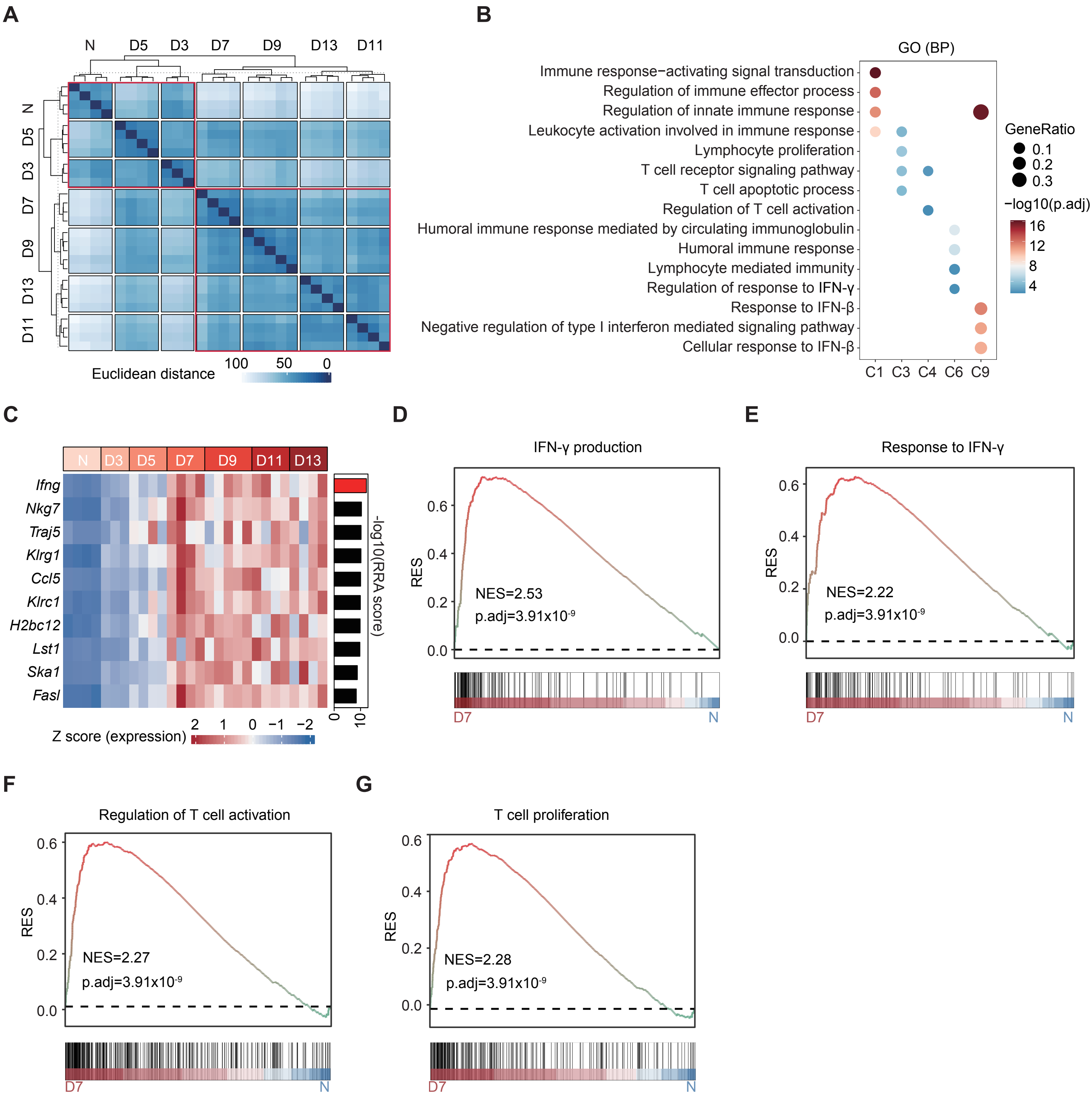
A
N
D5
D3
D7
D9
D13
D11
Euclidean distance
100
50
0
B
GO (BP)
Immune response−activating signal transduction
Regulation of immune effector process
Regulation of innate immune response
Leukocyte activation involved in immune response
Lymphocyte proliferation
T cell receptor signaling pathway
T cell apoptotic process
Regulation of T cell activation
Humoral immune response mediated by circulating immunoglobulin
Humoral immune response
Lymphocyte mediated immunity
Regulation of response to IFN-γ
Response to IFN-β
Negative regulation of type I interferon mediated signaling pathway
Cellular response to IFN-β
C1
C3
C4
C6
C9
GeneRatio
0.1
0.2
0.3
−log10(p.adj)
16
12
8
4
C
N
D3
D5
D7
D9
D11
D13
Ifng
Nkg7
Traj5
Klrg1
Ccl5
Klrc1
H2bc12
Lst1
Ska1
Fasl
-log10(RRA score)
0
10
Z score (expression)
2
1
0
−1
−2
D
IFN-γ production
RES
0.6
0.4
0.2
0.0
NES=2.53
p.adj=3.91x10-9
D7
N
E
Response to IFN-γ
RES
0.6
0.4
0.2
0.0
NES=2.22
p.adj=3.91x10-9
D7
N
F
Regulation of T cell activation
RES
0.6
0.4
0.2
0.0
NES=2.27
p.adj=3.91x10-9
D7
N
G
T cell proliferation
RES
0.6
0.4
0.2
0.0
NES=2.28
p.adj=3.91x10-9
D7
N

FIG S3. Time series transcriptomics analysis of the lungs of PbNK65-infected mice.

(**A**) Heterarchical clustering of the lung transcriptome based on the Euclidean distance. The two major clusters were boxed in red. (**B**) Gene Ontology (GO) analysis of genes in clusters C1, C3, C4, C6 and C9 in **FIG 2F**. (**C**) Heatmap of the expression level of the top 10 RRA-scored genes in C3 shown in **FIG 2G**. Expression levels were normalized using DESeq2 and represented as Z-scores, indicated by the color scale. The barchart in the right shows -log10(RRA scores) and the score of Ifng was highlighted in red. (**D-G**) Gene Set Enrichment Analysis (GSEA) for pathways related to IFN-γ and T cells.

FIG S4

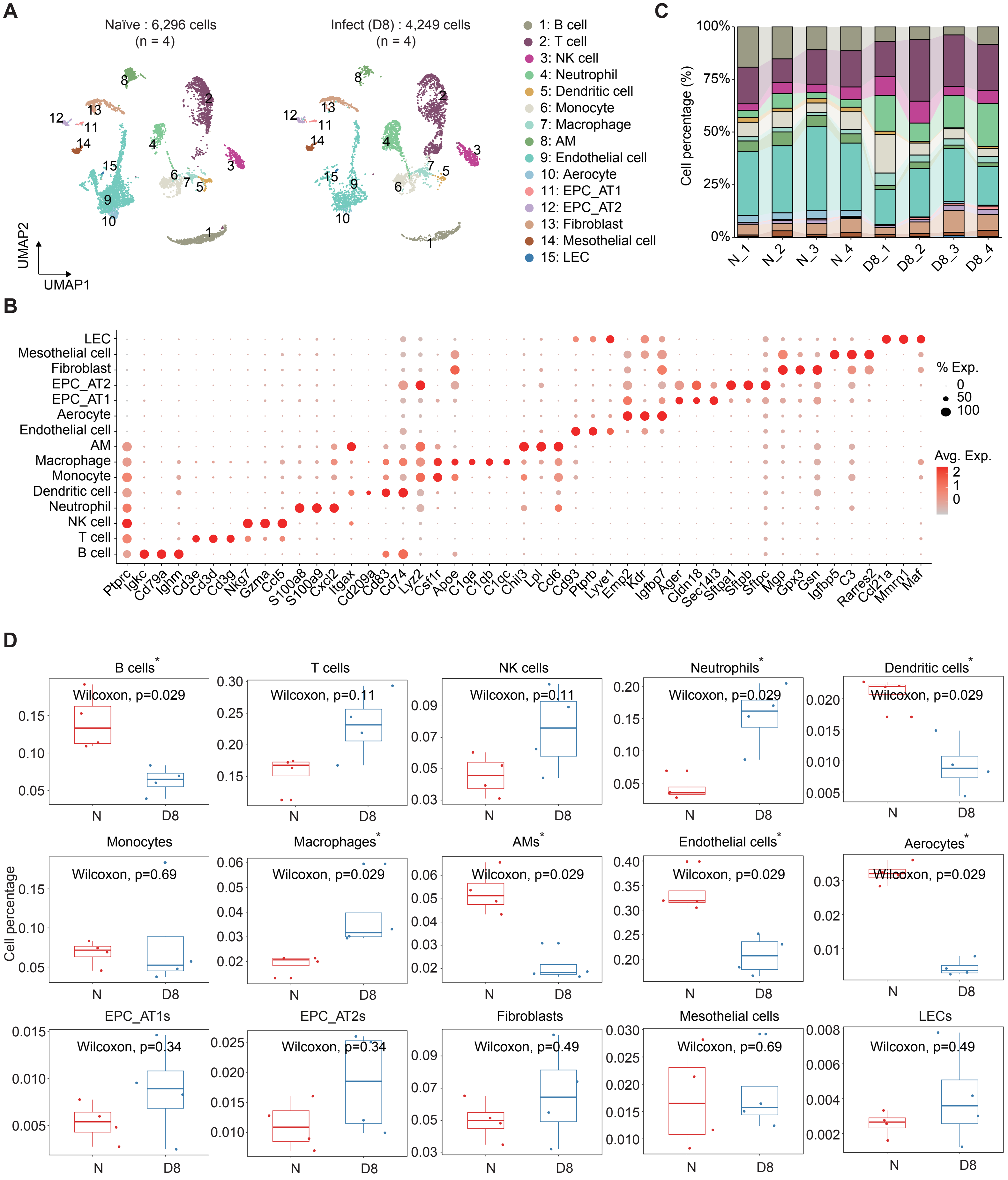
A
Naïve : 6,296 cells
(n = 4)
Infect (D8) : 4,249 cells
(n = 4)
UMAP1
UMAP2
1: B cell
2: T cell
3: NK cell
4: Neutrophil
5: Dendritic cell
6: Monocyte
7: Macrophage
8: AM
9: Endothelial cell
10: Aerocyte
11: EPC_AT1
12: EPC_AT2
13: Fibroblast
14: Mesothelial cell
15: LEC
C
Cell percentage (%)
100%
75%
50%
25%
0%
N_1
N_2
N_3
N_4
D8_1
D8_2
D8_3
D8_4
B
LEC
Mesothelial cell
Fibroblast
EPC_AT2
EPC_AT1
Aerocyte
Endothelial cell
AM
Macrophage
Monocyte
Dendritic cell
Neutrophil
NK cell
T cell
B cell
Ptprc Igkc Cd79a Ighm Cd3e Cd3d Cd3g Nkg7 Gzma Ccl5 S100a8 S100a9 Cxcl2 Itgax Cd209a Cd83 Cd74 Lyz2 Csf1r Apoe C1qa C1qb C1qc Chil3 Lpl Ccl6 Cd93 Ptprb Lyve1 Emp2 Kdr Igfbp7 Ager Cldn18 Sec14l3 Sftpa1 Sftpb Sftpc Mgp Gpx3 Gsn Igfbp5 C3 Rarres2 Ccl21a Mmrn1 Maf
% Exp.
0
50
100
Avg. Exp.
2
1
0
D
Cell percentage
B cells*
Wilcoxon, p=0.029
T cells
Wilcoxon, p=0.11
NK cells
Wilcoxon, p=0.11
Neutrophils*
Wilcoxon, p=0.029
Dendritic cells*
Wilcoxon, p=0.029
Monocytes
Wilcoxon, p=0.69
Macrophages*
Wilcoxon, p=0.029
AMs*
Wilcoxon, p=0.029
Endothelial cells*
Wilcoxon, p=0.029
Aerocytes*
Wilcoxon, p=0.029
EPC_AT1s
Wilcoxon, p=0.34
EPC_AT2s
Wilcoxon, p=0.34
Fibroblasts
Wilcoxon, p=0.49
Mesothelial cells
Wilcoxon, p=0.69
LECs
Wilcoxon, p=0.49
N
D8

FIG S4. Single-cell RNA-seq analysis of a mouse model of MA-ARDS.

(**A**) UMAP of lung cells isolated of naïve or infected mice colored by the cell-types. AM, alveolar macrophage; EPC_AT1/2, Type 1/2 alveolar epithelial cell; LEC, lymphatic endothelial cell. (**B**) Dot plot representing the expression level of the marker genes of each cell-types. The size of the symbols represents the proportion of cells expressing the marker gene, and the color intensity indicates its average expression level. (**C**) Stacked bar plot illustrating the distribution of the cell types in each naive (N) and infected (D8) samples. (**D**) Boxplots showing the relative abundance of different cell-types between the naive (N) and infected (D8) samples. P-values, Mann-Whitney t test. *, cell-types shown significant differences between the two groups.

FIG S5

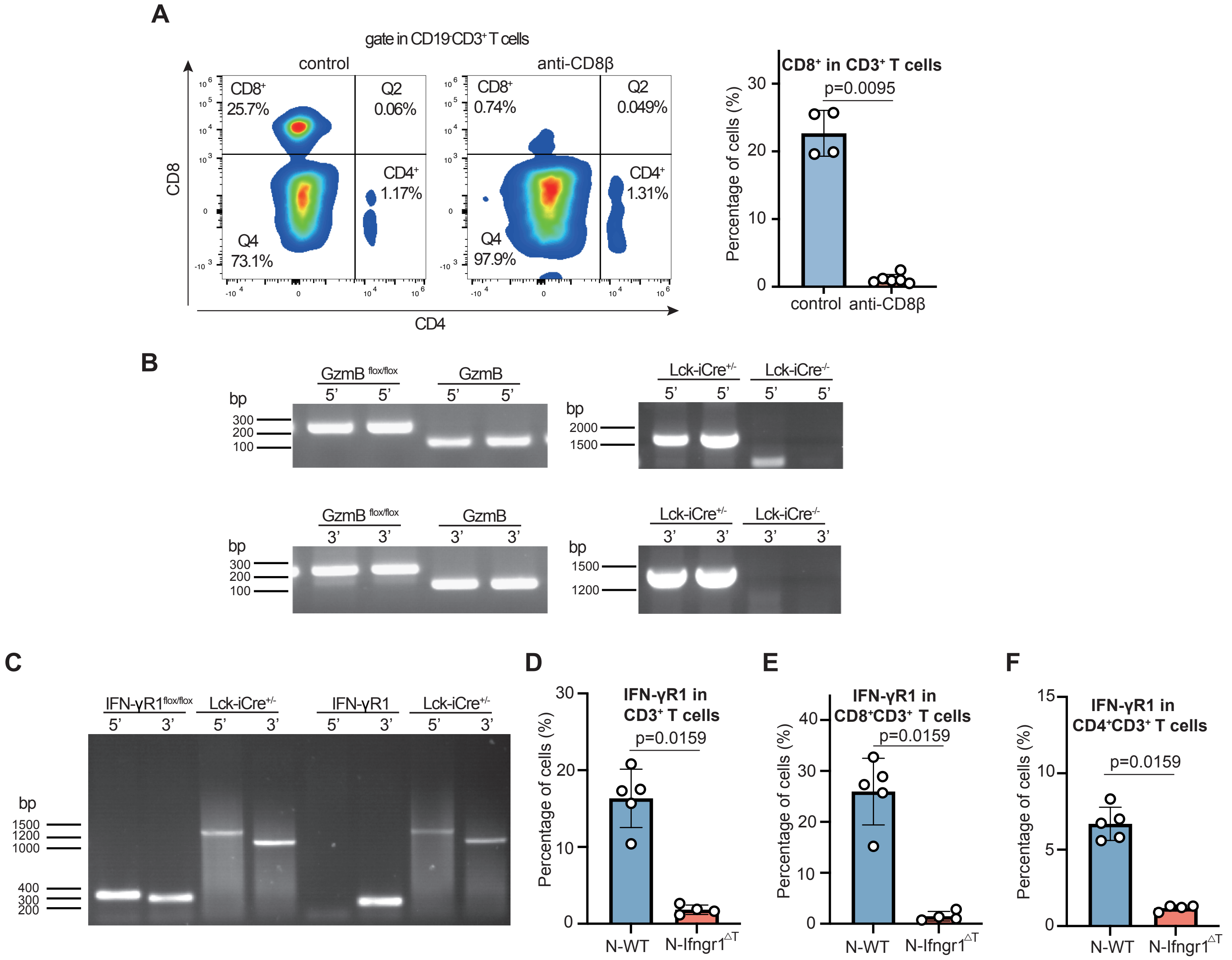

A
gate in CD19-CD3+ T cells
control
anti-CD8β
CD8+ 25.7%
Q2 0.06%
CD4+ 1.17%
Q4 73.1%
CD8+ 0.74%
Q2 0.049%
CD4+ 1.31%
Q4 97.9%
CD8
CD4
CD8+ in CD3+ T cells
p=0.0095
Percentage of cells (%)
control
anti-CD8β
B
GzmB flox/flox
GzmB
Lck-iCre+/-
Lck-iCre-/-
bp
300
200
100
2000
1500
1200
5'
3'
C
IFN-γR1flox/flox
Lck-iCre+/-
IFN-γR1
bp
1500
1200
1000
400
300
200
D
IFN-γR1 in CD3+ T cells
p=0.0159
E
IFN-γR1 in CD8+CD3+ T cells
p=0.0159
F
IFN-γR1 in CD4+CD3+ T cells
p=0.0159
Percentage of cells (%)
N-WT
N-Ifngr1ΔT

FIG S5. Genotyping of the conditional knockout mice and the knockout and depletion efficiency.

(**A**) Representative dot plot showing CD8 and CD4 expression on $CD19^{-}CD3^{+}$ T cells (left panel) and the percentage of $CD8^{+}$ T cells in $CD3^{+}$ T cells in the peripheral blood of the infected mice treated with anti-CD8β or isotype control antibodies. Mice were intravenously injected with 50 μg anti-CD8β antibodies or antibody isotype controls one day before intraperitoneal (i.p) injection of 10^{4} PbNK65-iRBCs. Two additional injections of 20 μg antibodies were performed at 4- and 8- dpi, and the peripheral blood was taken and assayed at 17 dpi. Each dot represents an individual mouse (n=4–6). P value, Mann-Whitney U test. (**B**) Genotyping of T cell-specific GzmB knockout ($GzmB^{\Delta T}$, $GzmB^{flox/flox}$ $lck\text{-}icre^{+/-}$) mice. WT, $lck\text{-}icre^{+/-}$ wildtype controls. Upper panels: 5', flox site or iCre site flanking region at 5' ends of gene; lower panels: 3', flox site or iCre site flanking region at 3' ends of gene. NS, not significant. (**C**) Genotyping of T cell specific Ifngr1 knockout ($Ifngr1^{\Delta T}$, Ifngr1 $^{flox/flox}$ $lck\text{-}icre^{+/-}$) mice. WT, lck-$icre^{+/-}$ wildtype controls. 5', flox site or iCre site flanking region at 5' ends of gene; 3', flox site or iCre site flanking region at 3' ends of gene. (**D-F**) Loss of IFN-γR1 expression on $CD3^{+}$ T cells in $Ifngr1^{\Delta T}$ mice (n=4–5). The percentages of IFN-$\gamma R1^{+}$ cells in total $CD3^{+}$ (**D**), $CD8^{+}CD3^{+}$ (**E**) and $CD4^{+}CD3^{+}$ (**F**) T cells in the spleens of naïve (N) mice. Each dot represents an individual mouse (n=4–5). Error bar, SD. P values, Mann-Whitney U test in (D-F).

FIG S6

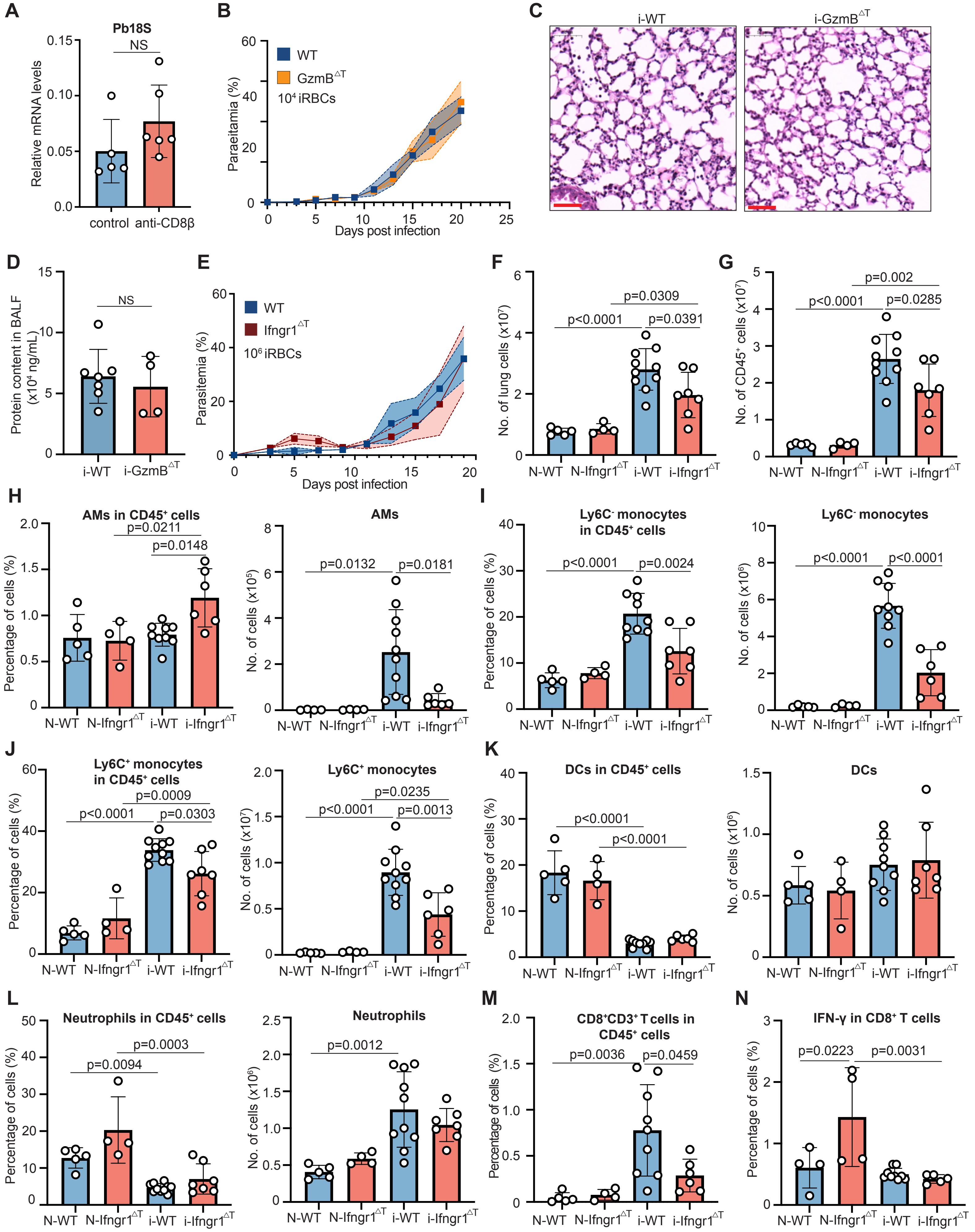

A
Pb18S
NS
Relative mRNA levels
control
anti-CD8β
B
WT
GzmB$^{\Delta T}$
10^4 iRBCs
Paraeitamia (%)
Days post infection
C
i-WT
i-GzmB$^{\Delta T}$
D
NS
Protein content in BALF ($\times 10^4$ ng/mL)
i-WT
i-GzmB$^{\Delta T}$
E
WT
Ifngr1$^{\Delta T}$
10^6 iRBCs
Parasitemia (%)
Days post infection
F
p=0.0309
p<0.0001
p=0.0391
No. of lung cells ($\times 10^7$)
N-WT
N-Ifngr1$^{\Delta T}$
i-WT
i-Ifngr1$^{\Delta T}$
G
p=0.002
p<0.0001
p=0.0285
No. of CD45$^+$ cells ($\times 10^7$)
H
AMs in CD45$^+$ cells
p=0.0211
p=0.0148
Percentage of cells (%)
AMs
p=0.0132
p=0.0181
No. of cells ($\times 10^5$)
I
Ly6C$^-$ monocytes in CD45$^+$ cells
p<0.0001
p=0.0024
Percentage of cells (%)
Ly6C$^-$ monocytes
p<0.0001
p<0.0001
No. of cells ($\times 10^6$)
J
Ly6C$^+$ monocytes in CD45$^+$ cells
p=0.0009
p<0.0001
p=0.0303
Percentage of cells (%)
Ly6C$^+$ monocytes
p=0.0235
p<0.0001
p=0.0013
No. of cells ($\times 10^7$)
K
DCs in CD45$^+$ cells
p<0.0001
p<0.0001
Percentage of cells (%)
DCs
No. of cells ($\times 10^6$)
L
Neutrophils in CD45$^+$ cells
p=0.0003
p=0.0094
Percentage of cells (%)
Neutrophils
p=0.0012
No. of cells ($\times 10^6$)
M
CD8$^+$CD3$^+$ T cells in CD45$^+$ cells
p=0.0036
p=0.0459
Percentage of cells (%)
N
IFN-γ in CD8$^+$ T cells
p=0.0223
p=0.0031
Percentage of cells (%)

FIG S6. Alleviated Lung pathologies in the infected *Ifngr1*$^{\Delta T}$ mice but not *GzmB*$^{\Delta T}$ mice

(A) Relative mRNA level of *P. berghei* 18S in the lungs of infected mice treated with anti-CD8β or isotype control antibodies. Mice were intravenously injected with 50 μg anti-CD8β antibodies or antibody isotype controls one day before intraperitoneal injection of 10^4 PbNK65-iRBCs. An additional injection of 20 μg antibodies were performed at 4 dpi and the lung collection was performed at 8 dpi. Each dot represents an individual mouse (n=5–6). Mouse β-Actin was used as an internal control. NS, not significant, Mann-Whitney U test. (**B)** Parasitemias of T cell specific-*GzmB* (*GzmB*$^{\Delta T}$, *GzmB* $^{flox/flox}$ lck-icre$^{+/-}$) compared to wild-type controls (WT, lck-icre$^{+/-}$) infected with 10^4 PbNK65-iRBCs. The box symbols represent the means and SD were shown as the light shadow of red and blue. (**C**) Representative photomicrographs of H&E-stained lung sections of 10^4-PbNK65-infected (i) *GzmB*$^{\Delta T}$ and WT mice at 7 dpi (n=4–7). Scale bar, 50 μm. (**D**) Total protein content in the BALF of infected mice collected at 7 dpi (n=4–7). Each dot represents an individual mouse. Error bar, SD. NS, not significant, Mann-Whitney U test. (**E**) Parasitemias of T cell-specific IFN-γR1 knockout (*Ifngr1*$^{\Delta T}$, *Ifngr1* $^{flox/flox}$ lck-icre$^{+/-}$) and WT control mice infected with 10^6 PbNK65-iRBCs. The results were representative of 2 independent experiments (n=4–7). The box symbols represent the means and SD were shown as the light shadow of red and blue. The numbers of total cells (**F**) and CD45$^+$ cells (**G**) in the lungs of naïve (N) and infected (i) *Ifngr1*$^{\Delta T}$ mice compared to the wildtype controls (WT) at 8 dpi after infection of 10^4 PbNK65-iRBC (n=4–10). P-values, One-way ANOVA with a Post Hoc Tukey's HSD test in (F-N). The percentages in CD45$^+$ cells and absolute cell numbers of alveolar macrophages (AMs) (**H**), Ly6C$^-$ monocytes (**I**), Ly6C$^+$ monocytes (**J**), dendritic cells (DCs) (**K**) and neutrophils (**L**) in the lungs of WT or *Ifngr1*$^{\Delta T}$ mice (n=4–10). (**M**) The percentages of CD8$^+$CD3$^+$ T cells in CD45$^+$ lung cells (n=4–9). (**N**) The percentage of CD8$^+$ T cells expressing IFN-γ (n=4–9). The cells were stimulated by PMA and ionomycin with the presence of GolgiStop before intracellular staining.

FIG S7

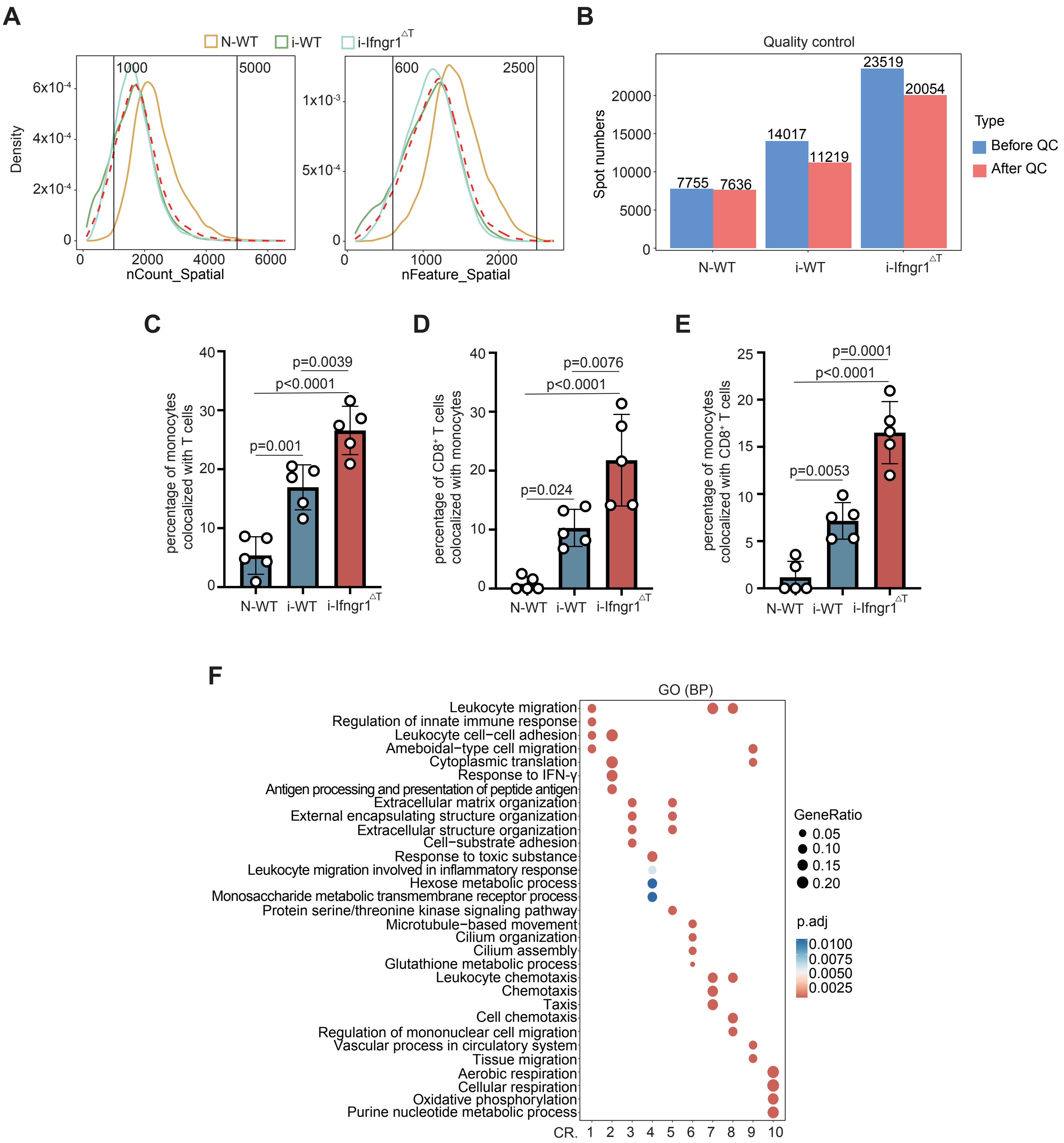

A
N-WT
i-WT
i-Ifngr1^ΔT
Density
nCount_Spatial
nFeature_Spatial
1000
5000
600
2500
B
Quality control
Spot numbers
Type
Before QC
After QC
7755
7636
14017
11219
23519
20054
N-WT
i-WT
i-Ifngr1^ΔT
C
percentage of monocytes colocalized with T cells
p=0.0039
p<0.0001
p=0.001
D
percentage of CD8+ T cells colocalized with monocytes
p=0.0076
p<0.0001
p=0.024
E
percentage of monocytes colocalized with CD8+ T cells
p=0.0001
p<0.0001
p=0.0053
F
GO (BP)
Leukocyte migration
Regulation of innate immune response
Leukocyte cell−cell adhesion
Ameboidal−type cell migration
Cytoplasmic translation
Response to IFN-γ
Antigen processing and presentation of peptide antigen
Extracellular matrix organization
External encapsulating structure organization
Extracellular structure organization
Cell−substrate adhesion
Response to toxic substance
Leukocyte migration involved in inflammatory response
Hexose metabolic process
Monosaccharide metabolic transmembrane receptor process
Protein serine/threonine kinase signaling pathway
Microtubule−based movement
Cilium organization
Cilium assembly
Glutathione metabolic process
Leukocyte chemotaxis
Chemotaxis
Taxis
Cell chemotaxis
Regulation of mononuclear cell migration
Vascular process in circulatory system
Tissue migration
Aerobic respiration
Cellular respiration
Oxidative phosphorylation
Purine nucleotide metabolic process
CR.
GeneRatio
p.adj

FIG S7. Spatial transcriptomics analysis.

(**A**) Quality control (QC) results of spatial transcriptomics data of the lungs of N-WT (naïve wildtype mouse), i-WT (infected wildtype mouse) and i-Ifngr1$^{\Delta T}$ (infected *Ifngr1*$^{\Delta T}$ mouse). The left panel displays the total number of genes detected per spot, and the right panel shows the number of unique genes detected per spot across the samples. Vertical lines indicate threshold values for QC. (**B**) Bar plot showing the number of spots before (blue) and after (red) QC in the 3 samples. The percentages of monocytes (CD11b$^+$) colocalized with T cells (CD3$^+$) (**C**) or CD8$^+$CD3$^+$ T cells (**E**), and CD8$^+$CD3$^+$ T cells colocalized with monocytes (**D**) in 20 FOVs (282 $\times$ 176 μm^2, avoiding big air tubes and blood vessels) randomly selected in the lung sections of the infected mice at 8 dpi (n=5). Error bar, SD; P-values, One-way ANOVA with a Post Hoc Tukey's HSD test. (**F**) Enrichment analysis of highly expressed genes in 10 region clusters identified in the spatial transcriptomic data. Genes with log2 (fold change) > 0 and Bonferroni-adjusted p-value < 0.05 were used for the analysis.

FIG S8

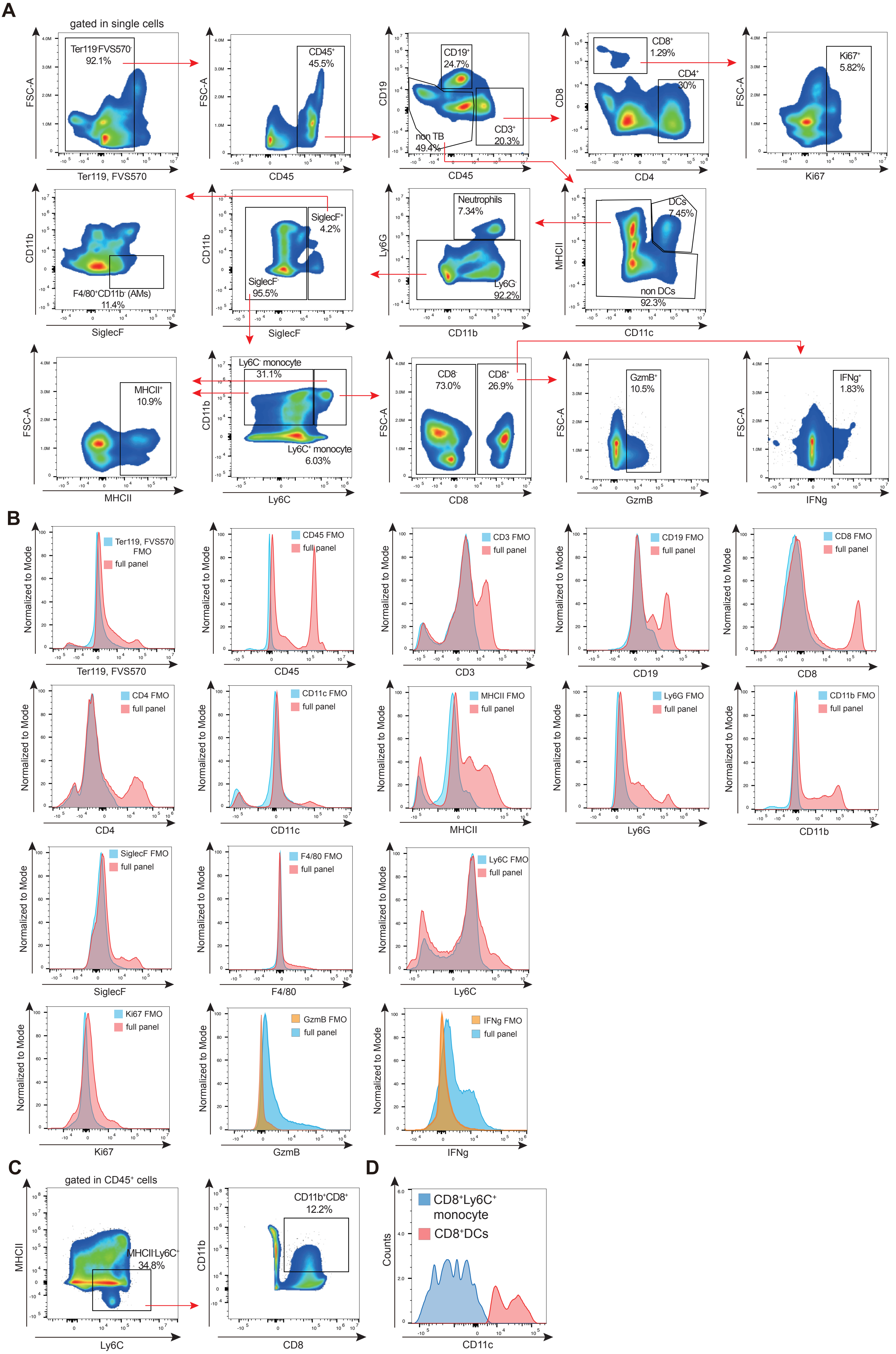
A
gated in single cells
Ter119⁻FVS570⁻
92.1%
FSC-A
Ter119, FVS570
CD45⁺
45.5%
CD45
CD19⁺
24.7%
non TB
49.4%
CD3⁺
20.3%
CD19
CD8⁺
1.29%
CD4⁺
30%
CD8
CD4
Ki67⁺
5.82%
Ki67
F4/80⁺CD11b⁻ (AMs)
11.4%
CD11b
SiglecF
SiglecF⁺
4.2%
SiglecF⁻
95.5%
Neutrophils
7.34%
Ly6G⁻
92.2%
Ly6G
CD11b
DCs
7.45%
non DCs
92.3%
MHCII
CD11c
MHCII⁺
10.9%
Ly6C⁻ monocyte
31.1%
Ly6C⁺ monocyte
6.03%
Ly6C
CD8⁻
73.0%
CD8⁺
26.9%
CD8
GzmB⁺
10.5%
GzmB
IFNg⁺
1.83%
IFNg
B
Normalized to Mode
Ter119, FVS570 FMO
full panel
CD45 FMO
CD3 FMO
CD19 FMO
CD8 FMO
CD4 FMO
CD11c FMO
MHCII FMO
Ly6G FMO
CD11b FMO
SiglecF FMO
F4/80 FMO
Ly6C FMO
Ki67 FMO
GzmB FMO
IFNg FMO
CD45
CD3
CD19
CD8
CD4
CD11c
MHCII
Ly6G
CD11b
SiglecF
F4/80
Ly6C
Ki67
GzmB
IFNg
C
gated in CD45⁺ cells
MHCII⁻Ly6C⁺
34.8%
MHCII
Ly6C
CD11b⁺CD8⁺
12.2%
CD11b
CD8
D
CD8⁺Ly6C⁺ monocyte
CD8⁺DCs
Counts
CD11c

FIG S8. Gating strategy and FMOs.

(**A**) Gating strategies used in the flow cytometry analysis shown in **FIG 3**, **FIG 5** and **FIG S6**. (**B**) FMO (Fluorescence minus one) controls for all antibodies used in lung intracellular staining panel (antibodies listed in lung panel 5 in TABLE S1). (**C**) The presence of $CD11b^{+}CD8^{+}$ cells gated in $CD45^{+}MHCII^{-}$ $Ly6C^{+}$ monocytes. (**D**) Histogram of CD11C expression on $CD8^{+}Ly6C^{+}$ monocytes (blue) $CD8^{+}$ DCs (red).

FIG S9

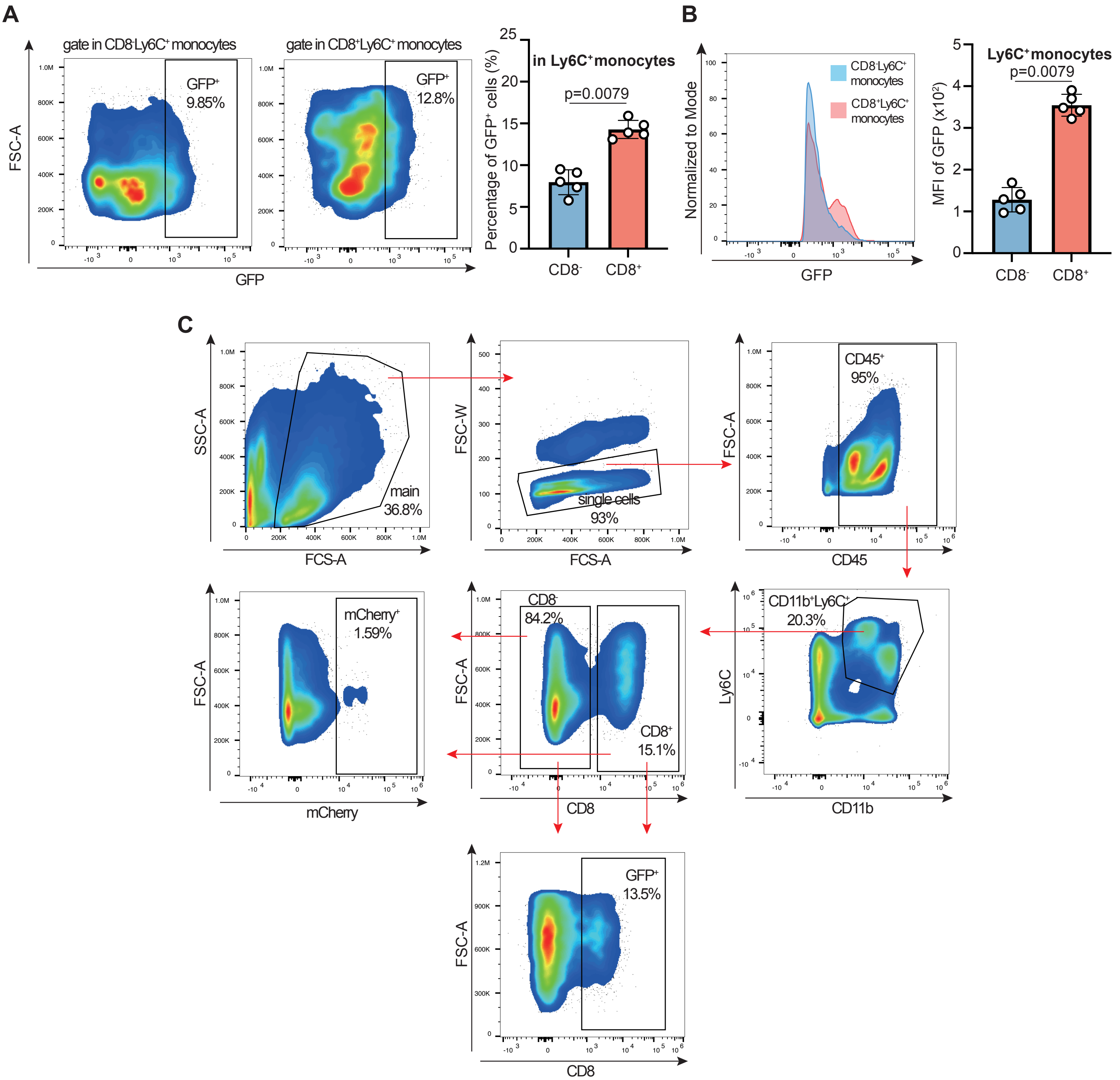
A
gate in CD8⁻Ly6C⁺ monocytes
gate in CD8⁺Ly6C⁺ monocytes
GFP⁺ 9.85%
GFP⁺ 12.8%
FSC-A
GFP
in Ly6C⁺monocytes
p=0.0079
Percentage of GFP⁺ cells (%)
CD8⁻
CD8⁺
B
CD8⁻Ly6C⁺ monocytes
CD8⁺Ly6C⁺ monocytes
Normalized to Mode
GFP
Ly6C⁺monocytes
p=0.0079
MFI of GFP (x10²)
CD8⁻
CD8⁺
C
SSC-A
FCS-A
main 36.8%
FSC-W
single cells 93%
CD45⁺ 95%
CD45
CD11b⁺Ly6C⁺ 20.3%
Ly6C
CD11b
CD8⁻ 84.2%
CD8⁺ 15.1%
CD8
mCherry⁺ 1.59%
mCherry
GFP⁺ 13.5%

FIG S9. $CD8^+Ly6C^+$ monocytes show enhanced phagocytic ability.

(**A**) Representative flow cytometry plots and percentages of $CD8^-Ly6C^+$ monocytes and $CD8^+Ly6C^+$ monocytes phagocyted GFP-expressing *E. coli* (n=5). *E. coli* and lung cells separated from 10^4-PbNK65-infected mice at 8 dpi were co-cultured at a ratio of 10:1 for 90 min. Each dot represents an individual mouse. Error bar, SD. P-values, Mann-Whitney U test in (A-B). (**B**) Mean of fluorescence intensity (MFI) of GFP in $CD8^-Ly6C^+$ monocytes (blue) and $CD8^+Ly6C^+$ monocytes (red) after co-culture (n=5). (**C**) Gating strategy used in the *in vitro* and *in vivo* phagocytosis assay.
